# Impact of universal interventions on social inequalities in physical activity among older adults: an equity-focused systematic review

**DOI:** 10.1186/s12966-017-0472-4

**Published:** 2017-02-10

**Authors:** Gesa Lehne, Gabriele Bolte

**Affiliations:** 1 Department of Social Epidemiology, Institute for Public Health and Nursing Research, University of Bremen, Grazer Strasse 2a, Bremen, 28359 Germany; 2 Health Sciences Bremen, University of Bremen, Bremen, Germany

**Keywords:** Physical activity, Social inequalities, Interventions, Intervention-generated inequalities, Equity impact assessment, Older adults

## Abstract

**Background:**

Physical activity is one of the most important contributors to healthy aging. Public health strategies aiming to promote physical activity among older adults are increasingly being implemented. However, little is known about their impact on social inequalities. Purpose of the study was to analyze whether and how studies of interventions consider effects on social inequalities in physical activity among older adults.

**Methods:**

Nine electronic databases were searched to identify quantitative studies evaluating the effects of interventions on self-reported or objectively measured physical activity among the general population of older adults (≥50 years). English and German language peer-reviewed journal articles published between 2005 and 2015 were included. Using the PROGRESS-Plus framework, data on whether and how social factors were considered both for describing participants’ baseline characteristics and for measuring intervention effects were systematically extracted. Studies examining differential intervention effects by at least one PROGRESS-Plus factor were quality assessed. Results were presented in narrative synthesis.

**Results:**

Fifty-nine studies were included. Beside age and sex, 44 studies used at least 1 further PROGRESS-Plus factor for the description of participants’ baseline characteristics. When measuring intervention effects, 22 studies considered PROGRESS-Plus factors as control variables. Eleven studies reported having analyzed potential effects on inequalities by testing interaction effects, stratifying effect analyses, or exploring associations between PROGRESS-Plus factors and increases in physical activity following an intervention. Effects were most often analyzed by gender/sex (*n* = 9) and age (*n* = 9), followed by education (*n* = 3), marital status (*n* = 2), and race/ethnicity (*n* = 2). Five studies pointed to gender/sex- or age-specific intervention effects, indicating that some interventions affect males and females, and younger and older individuals differently.

**Conclusions:**

Many studies evaluating the effects of interventions on physical activity among older adults have not exploited the potential for assessing effects on social inequalities so far. There is an urgent need for systematic application of appropriate methodological approaches and transparent reporting of social inequalities-related findings which can provide important indications for the design of those interventions most likely to be effective across all social groups of older adults.

**Trial registration:**

PROSPERO registration number: CRD42015025066

**Electronic supplementary material:**

The online version of this article (doi:10.1186/s12966-017-0472-4) contains supplementary material, which is available to authorized users.

## Background

Physical activity (PA) is one of the most important contributors to healthy aging [1]. Considerable evidence suggests that being sufficiently physically active has the potential to prevent major non-communicable diseases such as cardiovascular diseases, type 2 diabetes mellitus, obesity, cancer, depression, chronic respiratory diseases, dementia, and osteoporosis [2–4]. Despite the fact that regular PA is among the most important determinants of health and wellbeing, especially in older adults [5–7], epidemiological studies have shown that PA level tends to decline with increasing age [8, 9]. Evidence from social epidemiological studies, furthermore, indicates that the prevalence of sufficient PA differs between population subgroups such as those characterized by socioeconomic status (SES), race/ethnicity, or gender/sex [10–13]. With regard to older adults, low PA has been shown to be associated with female sex, low SES, living in a deprived residential area, low wealth, low education, not being white, not being married, and living alone [8, 14–18].

Interventions aiming to increase PA may be designed to specifically target the needs of socially disadvantaged population groups (i.e., particular subgroups represented by socioeconomic, sociocultural, and sociogeographical characteristics associated with social disadvantage [13]). These “targeted” interventions, if implemented successfully, may reduce inequalities in PA by increasing PA levels among socially disadvantaged population groups [13]. Moreover, “universal” (i.e., “non-targeted”) intervention strategies targeting the whole population are also described as a promising approach to tackle health inequalities. Kavanagh and colleagues [19] pointed out that universal intervention approaches have the potential to benefit larger numbers of people and can help to reduce inequalities within a population by disproportionally more benefiting socially disadvantaged population groups. However, there is a growing body of evidence suggesting that universal interventions, even if they are successful at improving health behaviors or health outcomes across the population, may widen social inequalities between different social groups [20–22]. These unintended effects are termed “intervention-generated inequalities” (IGIs) and may arise at any stage of the intervention process, from intervention provision, uptake, compliance, to outcome [23]. There is further evidence suggesting that IGIs are more likely to occur among interventions focusing on individual behavior changes (“downstream interventions”) compared to interventions focusing on social or policy changes (“upstream interventions”) [24].

For example, using systematic review methods, Hill and colleagues [25] have shown that increased tobacco price has the potential to reduce socioeconomic inequalities in smoking among adults. In contrast, non-targeted smoking cessation programs were found to have a negative impact on inequalities. The issue of IGIs has also been discussed in studies on obesity prevention interventions [26–30], interventions to promote healthy eating [31], school-based cognitive-behavioral [19], and school-based health behavior interventions [32]. In the area of PA promotion, Humphreys and Ogilvie [33] conducted a pilot systematic review analyzing how effects on social inequalities have been reported in systematic reviews and primary studies on environmental and policy interventions to promote PA. The authors found that, although relevant information (i.e., on participants’ baseline characteristics, adjusted associations, subgroup intervention effects, or interaction effects) was often provided within included studies, only few systematic reviews tended to synthesize intervention effects on social inequalities. In a recent systematic review of randomized controlled trials (RCTs), Attwood and colleagues [34] explored differences in the effects of primary care based PA interventions across indicators of social disadvantage among adults. They found a sufficient recording of information on indicators of social disadvantage allowing studies to analyze potential differences in intervention effects. However, since only few studies reported details of relevant analyses, firm conclusions regarding the impact of primary care based PA interventions on health inequalities could not be drawn.

Despite the fact that public health strategies aiming to increase PA among older adults are increasingly being implemented, it has, so far, not been systematically investigated whether these strategies have an impact on social inequalities. Nevertheless, the target population of older adults has been described as heterogeneous [35] containing various subgroups with diverse needs which may not be covered by a single intervention strategy. Consequently, some interventions may be differentially effective across social subgroups and thus may contribute to a widening of social inequalities in PA and PA-related health outcomes. In a recently published systematic review on the effectiveness of PA interventions for adults around the retirement age, Baxter and colleagues [36] discussed that only limited research had been conducted to assess potential inequalities in response to interventions. The authors reported little indication for differential effects between advantaged and disadvantaged population groups, without, however, giving detailed information on what evidence these conclusions were drawn. For the prioritization of those PA interventions most likely to be equally effective among older adults, a systematic investigation of the impact of these interventions on social inequalities, therefore, is urgently needed.

### Objectives

The objectives of this systematic review are to (1) describe the extent to which effects on social inequalities are considered in quantitative experimental and observational studies evaluating the effects of interventions on PA among the general population of older adults (≥50 years), (2) describe the methods used for measuring these effects, and (3) assess the implications of the social inequalities-related findings for health promotion research and practice.

## Methods

This systematic review was carried out following the PRISMA-Equity 2012 Extension for systematic reviews with a focus on health equity (PRISMA-E) [37, 38] (Additional file 1). It was registered with the PROSPERO international prospective register of systematic reviews (registration number: CRD42015025066), and the protocol has been published in *Systematic Reviews* [39]. To describe dimensions of social inequalities, the PROGRESS-Plus framework proposed by the Campbell and Cochrane Equity Methods Group [40] was used. The acronym PROGRESS represents eight dimensions across which inequalities may exist (*P*lace of residence, *R*ace/ethnicity/culture, *O*ccupation, *G*ender/sex, *R*eligion, *E*ducation, *S*ES, and *S*ocial capital [41]), and “Plus” considers other characteristics which may be associated with social disadvantage [42]. For the purpose of this review, SES was considered as a multidimensional concept (e.g., measured using multidimensional indices of objective SES or scales reflecting an individual’s perceived SES). Therefore, income was treated as a distinct aspect by adding it as a separate PROGRESS dimension. Place of residence was defined as using geographical aggregated SES measures representing the social and economic conditions of an individual’s neighborhood (e.g., using area level deprivation indices). Similar to SES, social capital was considered as a multidimensional concept (i.e., measured by using multidimensional indices). Finally, due to their association with health inequalities, age, marital status, and living situation (living alone versus living with others) were added as “Plus” characteristics. Studies considering effects on social inequalities were identified if authors reported differential effect analyses by at least one of the above defined PROGRESS-Plus factors. According to Kawachi et al. [43], health inequalities were considered as a descriptive term referring to any measurable differences in health between different social subgroups of a population without passing any moral judgement on the fairness or unfairness of these differences. In this sense, social inequalities in PA refer to any measurable differences in PA along PROGRESS-Plus factors.

### Search strategy

The search strategy was limited to English and German journal articles published since July 2005 and was applied in July 2015 to the following electronic databases: MEDLINE (via PubMed), PsycINFO (via Ovid), Cumulative Index to Nursing and Allied Health Literature (CINAHL) (via EBSCO Host), Cochrane Register of Controlled trials (CENTRAL) (via Cochrane Library), Physical Education Index (via ProQuest), Social Science Citation Index (SSCI) (via Web of Science), Applied Social Sciences Index and Abstracts (ASSIA) (via ProQuest), Sociological Abstracts (via ProQuest), and International Bibliography of the Social Sciences (IBSS) (via ProQuest). The search strategy (Additional file 2) comprised searching text words related to (1) physical activity, (2) interventions, (3) intervention effects, and (4) older adults in titles and abstracts. The reference lists of all studies analyzing differential intervention effects by at least one PROGRESS-Plus factor were examined to identify additional relevant articles. In addition, the German journal “Prävention und Gesundheitsförderung” was manually searched for further articles.

### Eligibility criteria

The review included peer-reviewed journal articles on studies reporting the effects of interventions on subjectively reported or objectively measured PA among adults aged 50 years and over. No restrictions on intervention characteristics or follow-up duration were applied, and also multi-component interventions were considered, irrespective of whether or not promoting PA was the main focus. All types of quantitative experimental and observational study designs, with and without control group, were eligible, with the exception of cross-sectional studies, unless the intervention was compared with a control condition. Eligible studies were those reporting on interventions targeting the general population of older adults, that is, potentially addressing everyone across the social spectrum (universal interventions). Moreover, beside participants’ age, eligible studies had to report characteristics of participants stratified by at least one PROGRESS-Plus factor.

Excluded were studies reporting on interventions designed to specifically target particular social groups of older adults. Furthermore, studies whose study participants, as a result of the studies’ inclusion and exclusion criteria, were restricted with regard to their actual PA behavior, functional status, weight status, or specific underlying medical conditions (e.g., criterion for inclusion in study was being “insufficiently active”, “functionally impaired”, “overweight”, or “having dementia”) were also excluded, as were studies focused on participants receiving nursing or rehabilitation care. Also excluded were studies that exclusively reported intervention effects on psychological outcomes (e.g., intentions, self-efficacy, attitudes) or physical function measures (e.g., muscle function, flexibility, gait speed).

### Study selection

An EndNote (ENDNOTE X7.1, Thomson Reuters) database was created to store all records retrieved. After removing duplicates, titles and abstracts were initially screened for eligibility by the first author. Two further reviewers each screened half of all identified records. Thus, final decisions on eligibility were based on consensus between two reviewers. Disagreements regarding eligibility were resolved through discussion or by consulting the last author. Full texts of all potentially eligible articles were assessed for final inclusion by the first author with a 20% random sample checked by the last author.

The strength of agreement between the reviewers was moderate (kappa value of 0.54) at the title and abstract stage, and substantial (kappa value of 0.70) at the full text stage.

### Data extraction and quality assessment

For the purpose of this review, a two-stage approach for data extraction was applied. At stage one, information on bibliographic details were extracted from all included studies as well as on study design, study aim(s), study participants, main intervention characteristics, and PA outcome(s). In order to classify studies based on their usage of PROGRESS-Plus factors, the data extraction form further captured information on whether and how PROGRESS-Plus factors were considered for the description of participants’ baseline characteristics and for measuring intervention effects. The latter was further differentiated according to whether PROGRESS-Plus factors were considered as control variables (e.g., by adjusting in multivariate analyses) or for analyzing differential intervention effects (e.g., by analyzing intervention effects stratified by categories of a PROGRESS-Plus factor or testing interactions between PROGRESS-Plus factors and interventions). At stage two, for studies examining differential intervention effects by at least one PROGRESS-Plus factor, an expanded data extraction form was applied capturing details on the methods used for measuring differential intervention effects as well as on PA outcome data from both overall and differential effect analyses. Stage one of data extraction was conducted by the first author and checked for accuracy by the last author in case of uncertainties. Stage two of data extraction was conducted by the first author and fully checked for accuracy by the last author.

The methodological quality of all studies included in stage two was appraised by both authors independently with any discrepancies resolved through discussion. A four-level scale of suitability of study design and a modified six-item checklist with methodological quality criteria were used, both previously proposed by Ogilvie et al. [44] (Additional file 3 A). These instruments were adapted by Ogilvie et al. [44] from the criteria used for the Community Guide of the US Task Force on Community Preventive Services [45] and for the Effective Public Health Practice Project in Canada [46]. Accordingly, each study was assigned to one of four categories, with studies including at least one before and one after measurement as well as a control group defined as most suitable. The checklist included the following six methodological quality criteria: Representativeness, Randomization, Comparability, Credibility of data collection instruments, Attrition rate, and Attributability to intervention. Both the suitability of study design and the methodological quality criteria were used for descriptive purposes as well as to highlight variations between studies and assess their validity.

### Data synthesis

Owing to the heterogeneity in the studies’ methods, a quantitative synthesis of review results (i.e., meta-analysis) was considered inappropriate. Instead, a narrative synthesis was conducted using a two-stage approach. At stage one, numbers of all included studies using PROGRESS-Plus factors for the description of participants’ baseline characteristics and for measuring intervention effects (i.e., using PROGRESS-Plus factors as control variables, and/or using PROGRESS-Plus factors for measuring differential intervention effects) were quantified by each PROGRESS-Plus factor separately. At stage two, studies that examined differential intervention effects by at least one PROGRESS-Plus factor were narratively presented, including tables containing information on significant study, sample and intervention characteristics, results from overall effect analyses, as well as on the methods and results of differential effect analyses.

## Results

**Fig. 1:**
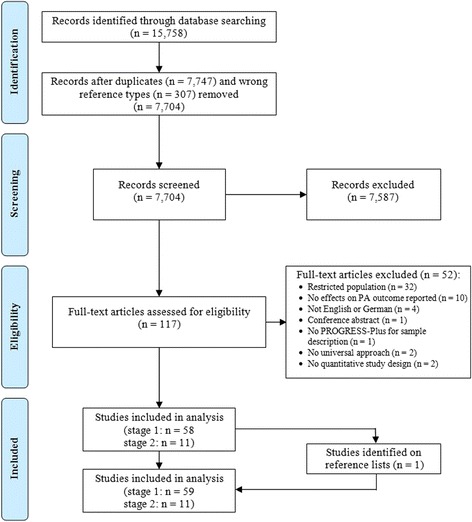
Flow diagram of study selection. The diagram illustrates the paper selection process containing number of identified records, included and excluded records, and the reasons why records were excluded. The diagram was adapted from the PRISMA statement [62]

The electronic database search identified 15,758 records which were reduced to 7704 after removal of duplicates and inappropriate reference types. After screening titles and abstracts, full texts of 117 potentially eligible articles were retrieved for in-depth review. Of those, 52 articles were excluded, mostly because they reported on studies with study populations restricted to particular subgroups of older adults. Of the remaining 58 studies (reported in 65 articles) (stage one), 11 were identified as having examined differential intervention effects by at least 1 PROGRESS-Plus factor (stage two). Screening the references cited in these 11 studies identified 1 additional study (reported in 1 article). Consequently, 59 studies (reported in 66 articles) were finally included in the review (stage one), among which 11 were considered for in-depth analysis (stage two) (Fig. 1). Main characteristics of all 66 articles are available as Additional file 4.

### Usage of PROGRESS-Plus factors

Almost all (*n* = 58) studies reported the age and gender/sex distribution of study participants (Table 1). The majority of studies (*n* = 44) additionally used at least one further PROGRESS-Plus factor for the description of participants’ baseline characteristics. Among these, education was the most commonly reported factor (*n* = 32), followed by race/ethnicity (*n* = 22), and marital status (*n* = 21). Twenty-two studies considered at least one PROGRESS-Plus factor as control variable when measuring intervention effects (e.g., by adjusting in multivariate analyses). Again, age (*n* = 22) and gender/sex (*n* = 19) were the factors most commonly controlled for, followed by education (*n* = 9). Among the 11 studies that used at least 1 PROGRESS-Plus factor for examining differential intervention effects, effects were most often analyzed by gender/sex (*n* = 9) and age (*n* = 9), followed by education (*n* = 3), marital status (*n* = 2), and race/ethnicity (*n* = 2).

**Table 1:** Usage of PROGRESS-Plus factors within all studies (*n* = 59)

PROGRESS-Plus factor	Use of PROGRESS-Plus factors		
	Sample description	Intervention effects	
		Control variables^a^	Differential effects
Place of residence	2	1	0
Race/ethnicity	22	2	2
Occupation	10	2	0
Gender/sex	58	19	9
Religion	1	0	0
Education	32	9	3
Socioeconomic status (SES)	2	0	0
Income	13	1	0
Social capital	1	0	0
Age	58	22	9
Marital status	21	3	2
Living situation	10	2	0
Total studies	59	22	11

^a^In 5 studies represented in the column, PROGRESS-Plus factors were considered as confounding factors, but not included in final analyses (Place of residence *n* = 1, Gender/sex *n* = 3, Education *n* = 1, Age *n* = 5, Living situation *n* = 1)

### Studies examining differential intervention effects by PROGRESS-Plus

Main characteristics of the 11 [47–57] studies using PROGRESS-Plus factors for examining differential intervention effects are summarized in Tables 2 and 3. All studies were conducted in developed countries. Ten studies [47–50, 52–57] used a longitudinal design with at least one before and one after measurement. Among these, six [47, 52–55, 57] included a control group and therefore had the highest suitability of study design. The remaining four studies [48–50, 56] used a single group pre-post design. For these 10 studies, the length of study follow-up varied from 1 month to 5 years. One study [51] used a cross-sectional design comparing an intervention with a control condition to explore intervention effects. This study could not be evaluated concerning its suitability since none of the four categories included in the scale of suitability of study design captured its design features adequately. Three studies [51–53] met five of the six quality criteria, four studies [49, 54, 55, 57] met four, three studies [48, 50, 56] met three, and the remaining study [47] met two quality criteria (Additional file 3 B).

**Table 2:** Characteristics of studies included in analysis stage 2 (*n* = 11)

Study	Quality	Location	Study design	Sample characteristics	Intervention	Physical activity outcome
Longitudinal study designs with two or more comparison groups						
Van Stralen et al. (2010) [57]	A, 4	Netherlands	Cluster RCT, IG1 (2 MHC) *n* ^a^ = 428, IG2 (2 MHC) *n* = 455, CG (2 MHC) *n* = 465 Follow-up = 12 months	≥50 years, community dwelling adults	IG1: 3 tailored letters; personalized PA advice targeting psychosocial determinants during 4 months IG2: IG1 plus tailored environmental information CG: Waiting-list	Self-report: Dutch SQUASH (total weekly min of PA, transport cycling, transport walking, leisure cycling, leisure walking, gardening, doing odd jobs, sports)
Peels et al. (2013) [54]	A, 4	Netherlands	Cluster RCT, IG1 (1 MHC) *n* ^a^ = 275, IG2 (1 MHC) *n* = 256, IG3 (2 MHC) *n* = 214, IG4 (1 MHC) *n* = 193, CG (1 MHC) *n* = 310 Follow-up = 12 months	≥50 years, community dwelling, sufficient understanding of Dutch language	IG1: 3 tailored letters; personalized PA advice targeting psychosocial determinants during 4 months IG2: IG1 plus tailored environmental information IG3: Web-based version of IG1 IG4: Web-based version of IG2 CG: Waiting-list	Self-report: Dutch SQUASH (total weekly days of sufficient PA (≥30 min), total weekly min of moderate to vigorous PA)
Harris et al. (2015) [52]	A, 5	UK	Cluster RCT, IG (118 households) *n* ^a^ = 142, CG (117 households) *n* = 138 Follow-up = 3 months	60–74 years, general practice registered patients, no contra-indications to increase PA	IG: 4 tailored primary care nurse delivered PA consultations over 3 months, pedometer and accelerometer feedback, individual PA diary and plan CG: Usual care	Objective: Accelerometer (average daily step-count)
Poulsen et al. (2007) [55]	A, 4	Denmark	Prospective controlled randomized follow-up study, IG (17 municipalities) *n* ^a^ = 997, CG (17 municipalities) *n* = 916 Follow-up 4.5 years	75- and 80-years, non-institutionalized	IG: Preventive home visits as part of daily routine in primary care plus education of home visitors over 3 years, group-based education of GPs CG: Preventive home visits	Self-report: Frequency of PA (‘high’ if >2×/month, ‘low’ if ≤ 1-2×/months)
Nahm et al. (2010) [53]	A, 5	USA	RCT, IG *n* ^b^ = 115, CG *n* = 100 Follow-up = 3 months	≥55 years, access to Internet/e-mail and able to use it independently, able to read and write English	IG: Social Cognitive Theory-based Structured Hip Fracture Prevention Website; learning modules, moderated discussion board, diaries CG: Conventional website	Self-report: Exercise dimension of the YPAS (weekly min of exercise)
Capodaglio et al. (2007) [47]	A, 2	Italy	Quasi experimental study, IG *n* ^b^ = 23, CG *n* = 15 Follow-up = 12 months	70–83 years, healthy, community-dwelling	IG: 1-year mixed strength training programme; 2×/week supervised exercise classes in hospital gym and 1×/week home sessions, encouragement of doing 30 min/week outdoor aerobic exercise CG: No intervention	Self-report: Paquap® (MDEE, aerobic activities >3 MET (AA3), PA intensity classes)
Longitudinal study designs with one group pre-post design						
Croteau & Richeson (2005) [48]	C, 3	USA	Before-and-after study, *n* ^a^ = 76 Follow-up = 4 months	60–90 years, living in congregate housing/community-dwelling, able to ambulate independently, no contraindications to PA	4-month community-based PA intervention (“A Matter of Health Walking Program”); goal setting, activity selection, self-monitoring, pedometer	Objective: Pedometer (daily step count)
Gellert et al. (2011) [50]	C, 3	Germany	Before-and-after study, *n* ^a^ = 302 Follow-up = 1 month	>60 years, no medical contraindications to PA	Intervention leaflet; goal setting, PA plan	Self-report: Adopted version of German-PAQ-50+ (weekly days of PA ≥30 min)
Fitzpatrick et al. (2008) [49]	C, 4	USA	Before-and-after study, *n* ^b^ = 418 Follow-up = 5-6 months	98% ≥60 years	4-month community-based PA intervention in senior centers; 16 chair exercises (strength, balance, flexibility, endurance), encouragement of walking, encouragement of doing exercise at home, pedometer	Self-report: Exercise items from SDSCA, 1998 BRFSS (daily min of PA)
Ståhl et al. (2013) [56]	C, 3	Sweden	Before-and-after study, *n* ^b^ = 195 Follow-up = 5 years	≥65 years	4-year outdoor environment intervention focused on improved accessibility/usability and safety/security as part of the “Let’s go for a walk” project	Self-report: Frequency of walking (within residential area) and of activity (within city area)
Cross-sectional study design with control group						
Hallgrimsdottir et al. (2015) [51]	NA, 5	Sweden	Cross-sectional study, IG *n* ^b^ = 358, CG *n* = 288	≥65 years	IG: See Stahl et al. [56] CG: Reference area without environmental intervention	Self-report: See Stahl et al. [56]

For quality assessment see Additional file 3

*Abbreviations*: *NA* Not applicable, *IG* Intervention group, *CG* Control group, *PA* Physical activity, *MHC* Municipal Health Councils, *SQUASH* Short Questionnaire to Assess Health-Enhancing Physical Activity, *GP* General practitioner, *YPAS* Yale Physical Activity Survey, *MDEE* Mean daily energy expenditure, *MET* metabolic equivalent, *German-PAQ-50+* German Physical Activity Questionnaire, *SDSCA* Summary of Diabetes Self-Care Activities, *BRFSS* Behavioral Risk Factor Surveillance System

^a^The numbers reported for sample size (*n*) correspond to the number of individuals included in analysis for measuring effects on PA

^b^The numbers reported for sample size (*n*) correspond to the number of individuals completing the study

**Table 3:** Methods and results of differential effects analyses of studies included in analysis stage 2 (*n* = 11)

Study	Overall intervention effect	Analysis of differential effects		
		PROGRESS-Plus	Methodological approach	Reported differential effects
Longitudinal study designs with two or more comparison groups				
Van Stralen et al. (2010) [57]	In1: No overall effects on PA. In2: Positive effect on total weekly min of PA.	Gender/sex, Education, Age, Marital status	Interaction terms between trial arms (CG as reference) and PROGRESS-Plus factors in multilevel linear regression model; stratification of data by categories of the PROGRESS-Plus factor for significant (*p* < 0.1) interaction terms and re-examining effects.	Significant In1 x age interaction (β_In1xage_ = −92.2; SD = 52.8; *p* = 0.08). In1: No significant effects for <65-year-olds and ≥65-year-olds. In2: Significant effect only in <65-year-olds (β_In2_ = 76.5; 95% CI = 4.9 to 148.0). No other significant interaction effects.
Peels et al. (2013) [54]	In1, In2: Positive effects on weekly days and min of PA. In3, In4: No overall effects on PA.	Gender/sex, Education, Age	Interaction terms between trial arms (CG as reference) and PROGRESS-Plus factors in multilevel linear regression model.	No significant interactions (*p* > 0.05).
Harris et al. (2015) [52]	Positive effects on average daily step-counts.	Gender/sex, Age	Interaction terms between trial arms and PROGRESS-Plus factors in multilevel regression model.	No significant interaction terms in regression model. Considering effect estimates with CI, significant effect in men (between group difference in change: 1534; 95% CI = 775–2294), no significant effect in women (591; 95% CI = −125 to 1307). Significant effects in all age groups (60–64, 65–69, 70–75 years).
Poulsen et al. (2007) [55]	NA–only subgroups presented.	Gender/sex and Age	Logistic regression analyses were stratified by gender/sex and age group.	Preventive home-visits: Significant effect on stabilizing PA in 80-year-old women. No effects in 75-year-old women and 75- and 80-year-old men. Educational intervention: Significant effect on increasing PA in 80-year-old women. No effects in 75-year-old women, and 75- and 80-year-old men.
Nahm et al. (2010) [53]	No overall effects on PA.	Gender/sex, Race/ethnicity, Age	Subgroup analyses with PROGRESS-Plus factors included as a second between subjects factor in mixed linear model.	No significant subgroup effects.
Capodaglio et al. (2007) [47]	Significant increase in AA3 time in intervention group (no change in control group). For remaining outcomes, only subgroups presented.	Gender/sex	Analyses (t-tests for dependent samples) were stratified by gender/sex group.	Significant effect (*p* < 0.05) on MDEE only in males; positive effects on Class 2 PA and AA3 time for both males and females, but different patterning of improvement. (No significant effects in control males and females).
Longitudinal study designs with one group pre-post design				
Croteau & Richeson (2005) [48]	Significant increase in daily step-counts.	Age	One-way ANOVA to test differences between age groups and improvement scores; LSD multiple comparison test following significant test result.	Significant difference in improvement scores between age groups (*p* = 0.028). Significantly larger effects for the youngest (60–64 years) age group compared to the 70–74, 75–79, 80–84 and ≥85 age groups. No significant difference between the youngest and 2nd youngest (65–69 years) age groups.
Gellert et al. (2011) [50]	Significant increase in PA.	Marital status	ANOVA of change to test differences in change in PA over time between partner status groups.	Significant time x partner status interaction (*p* < 0.05). Effects were stronger for participants whose partners also took part as compared to participants whose partner did not take part and participants without partner. No difference between the latter two subgroups.
Fitzpatrick et al. (2008) [49]	Significant increase in all PA outcomes except minutes of PA on physically active days.	Gender/sex, Race/ethnicity, Education, Age	Linear regression analyses to explore associations between PROGRESS-Plus factors and change in PA following the intervention.	No significant associations (*p* > 0.05).
Ståhl et al. (2013) [56]	No overall effects on PA.	Gender/sex, Age	Chi^2^-tests to test differences between gender/sex and age subgroups in change in PA following the intervention.	No significant differences.
Cross-sectional study design with control group				
Hallgrimsdottir et al. (2015) [51]	IG had significantly higher frequency of walking and activity compared to CG.	Gender/sex, Age	Interactions terms between PROGRESS-Plus factors and trial arms (CG as reference) in logistic regression model.	No significant interactions.

*Abbreviations*: *NA* Not applicable, *In* Intervention, *IG* Intervention group, *CG* Control group, *PA* Physical activity, *SD* Standard deviation, *CI* Confidence interval, *AA3* Aerobic activities over 3 metabolic equivalent (MET) intensity, *MDEE* Mean daily energy expenditure, *ANOVA* Analysis of variance, *LSD* Least Significant Difference

The content and intensity of interventions varied between studies, as did the level of intervention (i.e., individual, community), mode of delivery (e.g., PA sessions, face-to-face counseling, environmental improvements, interventions delivered by mail or the internet), and PA outcome measures, with self-reported measures most commonly used.

### Evidence synthesis on differential effect analyses by PROGRESS-Plus

Two cluster RCTs [54, 57] evaluated the effects of a larger intervention project aiming to increase PA among older adults in various municipalities in the Netherlands (Table 3). The first study [57] evaluated the effects of two sub-interventions each comprising three tailored letters with feedback on current PA level delivered over 4 months. The basic tailored intervention targeted psychosocial determinants alone, whereas the environmentally tailored additionally targeted environmental determinants. At 12 months, the latter sub-intervention was shown to be effective in increasing total weekly minutes of PA compared to the waiting-list control group. Effects on social inequalities were, methodologically, considered by testing interactions between trial arms (control group as reference) and the PROGRESS-Plus factors gender/sex, education, age, and marital status. A significant trial arm by age interaction was reported for the basic intervention. Subgroup analyses showed that the environmentally tailored intervention was only effective among younger (<65 years) but not effective among older (≥65 years) individuals, and that the basic intervention was equally ineffective for both younger and older participants. By gender/sex, education, and marital status, no significant interaction effects were found for neither intervention, suggesting that the environmentally tailored intervention was equally effective, and that the basic intervention was equally ineffective among males and females, lower, middle and higher educated, and single and married participants.

The second study [54] reported on a subsequent project phase in which both interventions were adapted and translated each into a web-based version. At 12 months, both printed sub-interventions were effective in increasing total weekly minutes of PA and weekly days of sufficient PA compared to the waiting-list control group, whereas both web-based sub-interventions were shown to be ineffective. Methodologically, effects on social inequalities were considered, just as in the previously reported study, by examining interactions between trial arms and PROGRESS-Plus factors, except that potential differences by marital status were not examined. None of the interactions tested were statistically significant suggesting that both printed interventions were equally effective, and both web-based interventions were equally ineffective, among males and females, lower and higher educated, and individuals of varying ages. However, (borderline) significant trial arm by age interactions for both printed interventions on weekly days of sufficient PA were reported at an earlier follow-up (6 months) [58] suggesting that the effects of both printed interventions on weekly days of sufficient PA favored older (≥65 years) compared to younger (50–64 years) participants. Moreover, significant trial arm by gender/sex interactions for the printed environmentally tailored intervention on minutes of PA and for the web-based environmentally tailored intervention on days of sufficient PA were reported, showing that the former was only effective in increasing minutes of PA in women, but not in men, and that the latter resulted in a decrease in days of sufficient PA in women, but in a non-significant increase in men.

A further cluster RCT [52] investigated the effects of a population-based primary care nurse-delivered complex intervention in older adults from three UK family practices. At intervention completion, the intervention group showed a greater increase in average daily step count as compared with the usual-care control group. Similar to both aforementioned studies, effects on social inequalities were considered by testing interactions between trial arms and PROGRESS-Plus factors. In this study, differences in effects were explored according to the participants’ gender/sex and age. The trial arm by age interaction was not significant showing that the intervention was equally effective among 60–64, 65–69, and 70–75-year-old participants. The trial arm by gender/sex interaction indicated a positive intervention effect in males but not in females.

A prospective controlled randomized follow-up study evaluated the effects of preventive home visits as well as of a 3-year educational intervention of home visitors and general practitioners among older residents in 34 Danish municipalities offering preventive home visits as part of the daily routine in primary care [55]. Methodologically, effects on social inequalities were considered using logistic regression analyses stratified by gender/sex and age group. Separately estimating effects among 75-year-old males, 80-year-old males, 75-year-old females, and 80-year-old females showed that preventive home visits were effective in stabilizing PA, and that the educational intervention was effective in increasing PA, but only among 80-year-old females. No positive effects of neither interventions were found for the 75-year-old women and the 75- and 80-year old men.

A RCT [53], preliminary evaluating the effects of a Social Cognitive Theory-based Structured Hip Fracture Prevention Website compared to a Conventional Website, reported having performed subgroup analyses to explore whether intervention effects differed by gender/sex, race/ethnicity, and age. After 3 months, the overall effect of the intervention on PA was shown to be null, and no indications for the presence of subgroup differences were observed.

In a quasi-experimental study [47], examining the effects of a mixed strength training program, effects on social inequalities were considered by testing change in PA over time among intervention and control group participants for males and females separately. A significant increase in mean daily energy expenditure was found only for males who received the intervention. Weekly hours of aerobic activities greater than three metabolic equivalents (MET) and of Class 2 PA (4–5.9 MET) increased over time in both sexes receiving the intervention, but patterns of improvements appeared to be different (males: >3 MET: +51%, Class 2 PA: +146%; females: +41%, +18%).

A longitudinal single group pre-post study [48] evaluated the effects of the community-based “A Matter of Health Walking Program” among older adults in Maine (USA), with pedometer-provided feedback on PA as main motivational tool. At intervention completion, a significant increase in daily step-counts was found. Effects on social inequalities were considered by testing whether improvement scores differed by age. Using analysis of variance (ANOVA), a significant difference between age group and improvement scores was found. Further exploring this difference suggested that the youngest age group (60–64 years) significantly greater increased its average daily steps compared to the 70–74, 75–79, 80–84, and ≥85 age groups, with an exception of a non-significant difference between the youngest and second youngest age group (65–69 years).

Using data collected in the context of a small-scale leaflet intervention to foster PA among a convenience sample of older adults in Germany, one single group pre-post study [50] analyzed the effects of social integration and exercise specific social support on PA. Overall, a significant increase in PA between baseline and 1-months follow-up was shown. Effects on social inequalities were considered for marital status by differentiating between three different partner status groups. Using ANOVA of change, a significant time by partner status interaction was found suggesting that time effects on PA differed by partner status. A substantially increase in PA was found only among participants whose partner took part in the intervention but not among participants who either were single or who had a partner that did not take part in the intervention.

Another before-and-after study [49] investigated the effects of a statewide community-based PA intervention comprising educator-led chair exercises, encouragement of walking, and using a pedometer among a convenience sample of older adults attending senior centers in Georgia (USA). Overall, significant increases for almost all PA outcomes were found. Effects on social inequalities were considered by exploring whether race/ethnicity, gender/sex, education, and age were associated with changes in PA following the intervention using linear regression analysis. No significant associations between PROGRESS-Plus factors and changes in PA were found suggesting that neither males nor white, higher educated, and younger individuals were more likely than females, black, lower educated, and older individuals, respectively, to increase their PA following the intervention.

As part of a larger intervention project called “Let’s go for a walk”, two studies [51, 56] evaluated the implementation of environmental measures focusing on accessibility/usability and safety/security in a geographically defined area in a medium-sized town in Sweden. Using a single group pre-post design, the first study [56] found no positive change in PA between baseline and 5-year follow-up. Methodologically, effects on social inequalities were considered by examining differences in PA outcomes between age and gender/sex subgroups using Chi^2^-tests. In these analyses, neither age nor gender/sex differences in change in PA were observed. In the second study [51], a cross-sectional design was used comparing the study area with a reference area in which no environmental changes were made. Overall, 5 to 8 years post-intervention, participants in the study area were significantly more physically active than participants in the reference area. To consider effects on social inequalities, interactions between participants’ age and gender/sex and the trial arms (i.e., areas) were explored using logistic regression analyses. No significant interactions were found suggesting that the environmental intervention did not change gender/sex and age patterns of PA.

## Discussion

### Main findings

This is the first systematic review that synthesized the evidence on whether and how effects on social inequalities are considered in quantitative studies evaluating the effects of interventions on PA among the general population of older adults. The results suggest that the majority of studies provided information on various social factors described by PROGRESS-Plus. When measuring intervention effects, however, analyses were most often designed to control for PROGRESS-Plus factors. Only a small number of studies (*n* = 11) reported having analyzed differential intervention effects by at least one PROGRESS-Plus factor. The methodological approaches applied for exploring differential intervention effects varied between studies and included, for example, adding interaction terms between PROGRESS-Plus factors and intervention variables in a multivariate analysis model, stratifying effect analyses by different categories of a PROGRESS-Plus factor, or exploring associations between PROGRESS-Plus factors and changes in PA following an intervention.

Overall, differential effect analyses were primarily oriented towards gender/sex and age comparisons, with mixed evidence for differential intervention effects across categories of both factors. Differences in intervention effects according to other dimensions of social inequalities, such as education, race/ethnicity, or marital status, were less frequently considered. For these factors, no indications for differential intervention effects were found.

### Comparison with other research

Up to now, little research has been conducted to assess the impact of public health strategies on social inequalities in PA, particularly with regard to interventions focusing on older adults. In a previous systematic review, Baxter and colleagues [36] examined the effectiveness of interventions to increase PA among adults around the time of retirement. Regarding effects on inequalities, the authors concluded that studies rarely reported having analyzed differential effects in subgroups of older adults, with little indication for differential effects between advantaged and disadvantaged population groups. However, since assessing the impact of interventions on social inequalities was not the main focus of the review, evidence supporting their conclusion regarding differential effects was not presented in greater depth.

The present systematic review extends the limited evidence regarding the impact of public health strategies on social inequalities in PA among older adults threefold. First, it systematically describes the extent to which intervention effects on social inequalities are considered, giving an impression of how many universal intervention studies provide data on various sociodemographic and socioeconomic factors described by PROGRESS-Plus but do not analyze, or at least do not report having analyzed, possible effects on social inequalities. Second, focusing on those studies that analyzed differential intervention effects by PROGRESS-Plus, it identifies the methods used for analyzing these effects. And third, it synthesizes the available evidence on potential effects on social inequalities.

Previously, two review studies have been conducted that aimed to synthesize the evidence on differential effects of PA interventions systematically and comprehensively using the PROGRESS-Plus framework [33, 34]. None, however, have focused especially on older adults or were designed to capture the evidence from all types of public health strategies that might impact on social inequalities in PA. Compared to Humphreys and Ogilvie [33], who found that over 40% of experimental and quasi-experimental studies on environmental and policy PA interventions reported subgroup effects and 18% interaction effects, the present review found that only 19% of studies reported having analyzed differential intervention effects. The rare evaluation of potential effects of PA interventions on social inequalities found in the present review is in line with findings of a systematic review by Attwood and colleagues [34] who found that 14% of RCTs on primary-care-based PA interventions reported differential effect analyses. Also in line with Attwood et al. [34], the present review further indicates that, where effects on social inequalities are considered, intervention effects are predominantly compared by gender/sex and age. There is evidence suggesting that other PROGRESS-Plus factors considered in this review, such as race/ethnicity, occupation, education, SES, marital status, and living situation, may also be associated with PA among older adults [8, 14, 17, 18]. Despite the fact that some of these factors (especially race/ethnicity, education, and marital status) were frequently measured, these factors were rarely or not considered at all when analyzing differential intervention effects. In their pilot systematic review, Humphreys and Ogilvie [33] also found that intervention effects were most often compared by gender/sex, whereas age differences, however, were comparatively less frequently analyzed. In the present review, mixed evidence for differential intervention effects for gender/sex and age were found supporting the presumption that different types of interventions might affect males and females as well as younger and older individuals differently. Gender differences in intervention effects were also reported by Humphreys and Ogilvie [33] and Attwood et al. [34]. However, no evidence for differential effects by age was found by both author groups.

### Future research

There is a need for studies on interventions aiming to promote PA among older adults to adequately conduct and report differential effect analyses. Information on the social distribution of intervention effects is a prerequisite for the design and implementation of interventions not increasing the health gap between different social groups or, better still, reducing social inequalities. It is often criticized that few studies have adequate sample sizes and diversity to allow for the conduct of appropriate differential effect analyses [59]. However, as mentioned by Moore and colleagues [32], the consistent reporting of subgroup intervention effects across studies, even if individual studies are not sufficiently powered to directly look at these effects, would allow for pooling effects across studies. This, in turn, would allow for investigating characteristics of interventions that are more or less effective among specific population subgroups or, just as important, that are likely to be equally effective across population subgroups.

Despite the fact that PROGRESS-Plus factors were frequently measured in studies included in the present review, only a minority had an explicit emphasis on analyzing differential effects suggesting that the potential for assessing effects on social inequalities has not been exploited. Against this background, there is a need for practical guidance on methods for adequate analysis and transparent reporting of differential effect analyses in evaluation studies. A promising project is the ongoing development of CONSORT-equity, an extension of the Consolidated Standards of Reporting Trials (CONSORT) guideline for health equity concerns in RCTs [60]. Given that RCTs are likely to be less frequently conducted in the field of public health and health promotion [61], consideration should also be given to the development or adoption of guidelines to suit the needs of study designs other than RCTs. Finally, examining the effectiveness of interventions specifically targeting certain social groups of older adults will further strengthen the evidence regarding the impact of interventions on social inequalities in PA among older adults and, therefore, represents an important topic for future systematic reviews.

### Strengths and limitations

The application of a comprehensive search strategy to capture a broad range of public health strategies, including studies operating at the individual, community, or societal level, is one strength of this review. Consideration was given to all types of study designs used to evaluate intervention effects on PA, with the exception of cross-sectional studies, unless the intervention was compared with a control condition. The resulting challenging heterogeneity in intervention characteristics, study designs, PA outcomes, and methodological approaches used to examine differential intervention effects was handled using narrative synthesis in conjunction with tabular illustrations. By limiting the inclusion criteria to English and German language peer-reviewed journal articles published between July 2005 and 2015, some potentially relevant studies, however, may have been missed.

Although previously used for assessing the quality of public health interventions [30, 33, 44], the scale of suitability of study design did not capture all study designs identified in the present review (one study could not be placed in any of the four categories). Furthermore, the significance of both the “Attrition rate” and “Attributability to intervention” methodological quality criteria turned out to be questionable. For example, studies reporting attrition rates of more than 30%, although applying additional sensitivity analyses to account for selective dropout, were not able to meet the “Attrition rate” criterion and had to be treated just as studies not containing any information on attrition rates. The judgement about the “Attributability to intervention” criterion was based upon whether or not a study explicitly mentioned that there was evidence of contamination of a control group, a concurrent intervention, or other contextual factors that could also have explained the observed effects. This criterion was met by all studies, since corresponding claims were not presented in neither study.

Considering a broad range of quantitative study designs means that not all studies included in the evidence synthesis were capable of examining *true* differential intervention effects (e.g., four studies had no control group, one study was cross-sectional in design). It should further be noted that most studies included in the evidence synthesis on differential effect analyses used self-reported measures of PA. All except one of these studies met the “Credibility of data collection instruments” quality criterion by showing that PA data collection tools were valid and reliable. However, whether the methods used for measuring intervention effects were valid in terms of their ability to detect behavioral change over time (i.e., intervention effects) is not reported. Finally, the review focused on studies of interventions potentially addressing everyone across the social spectrum. However, some of the included studies, although not explicitly focusing on particular subgroups of older adults, reported on rather selective study samples, possibly due to the studies’ recruitment strategies.

## Conclusions

The results of this systematic review suggest that many studies evaluating the effects of universal interventions on PA among older adults have not exploited the potential for assessing differential intervention effects across social groups so far. Currently, there is insufficient evidence to allow drawing firm conclusions regarding the impact of these interventions on social inequalities. The majority of studies, however, collected sufficient information on relevant characteristics described by PROGRESS-Plus to permit differential intervention effects to be examined. There is an urgent need for systematic application of appropriate methodological approaches as well as transparent reporting of social inequalities-related analyses and findings which can provide important indications for prioritization of those interventions most likely to be effective across all social groups of older adults.

## Additional files

Additional file 1: PRISMA-Equity 2012 Extension: Reporting Guidelines for Systematic Reviews with a Focus on Health Equity. This file provides a completed PRISMA-Equity 2012 checklist. (DOCX 23 kb)
Additional file 2: Sample search string for PubMed MEDLINE. This file contains a sample search string for PubMed MEDLINE. (DOCX 13 kb)
Additional file 3: A. Suitability of study design and methodological quality criteria. B. Results of Quality assessment. This file contains two tables providing information on the criteria used for assessing the methodological quality of studies (A) and on the results of the quality assessment (B). (DOCX 19 kb)
Additional file 4: Characteristics of articles (*n* = 66) reporting on studies (*n* = 59) included in analysis stage 1. This file contains a table in which characteristics of all included articles are summarized. (DOCX 123 kb)

## Abbreviations

ANOVA: Analysis of variance
ASSIA: Applied Social Science Index and Abstracts
CENTRAL: Cochrane Register of Controlled Trials
CINAHL: Cumulative Index to Nursing and Allied Health Literature
CONSORT: Consolidated Standards of Reporting Trials
CRD: Centre for Reviews and Dissemination
IBSS: International Bibliography of the Social Sciences
IGIs: Intervention-generated inequalities
MET: Metabolic equivalent
PA: Physical activity
PRISMA: Preferred reporting items for systematic reviews and meta-analyses
PRISMA-E: PRISMA-Equity 2012 Extension, Reporting Guidelines for Systematic Reviews with a Focus on Health Equity
PROGRESS-Plus: Place of residence, Race/ethnicity/culture, Occupation, Gender/sex, Religion, Education, Socioeconomic status, Social capital. “Plus” considers other categories that may impact on health equity
PROSPERO: International prospective register of systematic reviews
RCTs: Randomized controlled trials
SES: Socioeconomic status
SSCI: Social Science Citation Index
