# Supplementary material for: Impact of universal interventions on social inequalities in physical activity among older adults: an equity-focused systematic review
Source: Int J Behav Nutr Phys Act. 2017 Feb 10;14:20. doi: 10.1186/s12966-017-0472-4 (PMC5303302; doi:10.1186/s12966-017-0472-4)
Supplement: Additional file 3: — A. Suitability of study design and methodological quality criteria. B. Results of Quality assessment. This file contains two tables providing information on the criteria used for assessing the methodological quality of studies (A) and on the results of the quality assessment (B). (DOCX 19 kb) [file 12966_2017_472_MOESM3_ESM.docx]

**Additional file 3**

**A. Suitability of study design and methodological quality criteria (modified* from Ogilvie et al.)**

|  | **Description** |
| --- | --- |
| **Suitability of study design** | |
| Category A | The study design includes concurrent comparison groups AND prospective measurement of exposure and outcome. |
| Category B | The study design includes at least two ‘before’ measurements and at least two ‘after’ measurements but no concurrent comparison group. |
| Category C | The study design involves single ‘before’ and ‘after’ measurements with no concurrent comparison group. |
| Category D | The study design involves measurements of exposure and outcome made at a single point in time. |
| **Methodological quality criteria** | |
| Representativeness | Were the study samples randomly recruited from the study population with a response rate of at least 60% OR were they otherwise shown to be representative of the study population? |
| Randomization | Were participants, groups or areas randomly allocated to receive the intervention or control condition? |
| Comparability | Were the baseline characteristics of the comparison groups comparable OR if there were important differences in potential confounders were these appropriately adjusted for in the analysis? For studies without comparison group: Were important confounding factors appropriately controlled for in the analyses? |
| Credibility of data collection  instruments | Were data collection tools shown to be credible, e.g. shown to be valid and reliable in published research, OR in a pilot study, OR taken from a published national survey, OR recognized as an acceptable measure (such as biochemical measures of smoking). |
| Attrition rate | Were outcomes studied in a panel of respondents with an attrition rate of less than 30% OR were results based on a cross-sectional design with at least 200 participants included in analysis in each wave? |
| Attributability to intervention | Is it reasonably likely that the observed effects were attributable to the intervention under investigation? This criterion cannot be met if there is evidence of contamination of a control group in a controlled study. Equally, in all types of study, if there is evidence of a concurrent intervention that could also have explained the observed effects and was not adjusted for in analysis, this criterion cannot be met. |

* In the original version proposed by Ogilvie et al., the quality criterion “Comparability” can only be met by studies including a comparison group. For the present review, the criterion was modified so that it could also be met by uncontrolled studies appropriately controlling for possible confounding factors (i.e., by adjusting effect analyses).

**Reference**

Ogilvie D, Fayter D, Petticrew M, Sowden A, Thomas S, Whitehead M, et al. The harvest plot: a method for synthesising evidence about the differential effects of interventions. BMC Med Res Methodol. 2008;8:8.

**B. Results of Quality assessment**

| **Study** | Suitability of  study design | | | | **Methodological quality criteria** | | | | | |
| --- | --- | --- | --- | --- | --- | --- | --- | --- | --- | --- |
|  | A | B | C | D | Representativeness | Randomization | Comparability | Credibility of data collection instruments | Attrition rate | Attributability  to intervention |
| Van Stralen et al. [57] | 🗸 |  |  |  |  | 🗸 | 🗸 | 🗸 |  | 🗸 |
| Peels et al. [54] | 🗸 |  |  |  |  | 🗸 | 🗸 | 🗸 |  | 🗸 |
| Harris et al. [52] | 🗸 |  |  |  |  | 🗸 | 🗸 | 🗸 | 🗸 | 🗸 |
| Poulsen et al. [55] | 🗸 |  |  |  | 🗸 | 🗸 | 🗸 |  |  | 🗸 |
| Nahm et al. [53] | 🗸 |  |  |  |  | 🗸 | 🗸 | 🗸 | 🗸 | 🗸 |
| Capodaglio et al. [47] | 🗸 |  |  |  |  |  |  | 🗸 |  | 🗸 |
| Croteau & Richeson [48] |  |  | 🗸 |  |  |  |  | 🗸 | 🗸 | 🗸 |
| Gellert et al. [50] |  |  | 🗸 |  |  |  |  | 🗸 | 🗸 | 🗸 |
| Fitzpatrick et al. [49] |  |  | 🗸 |  |  |  | 🗸 | 🗸 | 🗸 | 🗸 |
| Ståhl et al. [56] |  |  | 🗸 |  | 🗸 |  |  | 🗸 |  | 🗸 |
| Hallgrimsdottir et al. [51] | NA | NA | NA | NA | 🗸 |  | 🗸 | 🗸 | 🗸 | 🗸 |

**Abbreviations:** NA = Not Applicable.
