# Supplementary material for: Impact of universal interventions on social inequalities in physical activity among older adults: an equity-focused systematic review
Source: Int J Behav Nutr Phys Act. 2017 Feb 10;14:20. doi: 10.1186/s12966-017-0472-4 (PMC5303302; doi:10.1186/s12966-017-0472-4)
Supplement: Additional file 4: — Characteristics of articles (n = 66) reporting on studies (n = 59) included in analysis stage 1. This file contains a table in which characteristics of all included articles are summarized. (DOCX 123 kb) [file 12966_2017_472_MOESM4_ESM.docx]

Additional File 4: Characteristics of articles (n=66) reporting on studies (n=59) included in analysis stage 1

| **Author (year)** | **Location** | **Study design** | **Sample characteristics (PROGRESS-Plus)** | **Approach** | **PA Outcome(s)** | **Use of PROGRESS-Plus** | |
| --- | --- | --- | --- | --- | --- | --- | --- |
|  |  |  |  |  |  | **Control variables** | **Differential effects** |
| **Longitudinal study designs with two or more groups** | | | | | | | |
| Azizan et al. (2013) [1] | Malaysia | Controlled quasi-experimental study,  IG1 *n*^d^=18, IG 2 *n*=23, CG *n*= 22 | *n*=63  ***Gender/sex***: % female  IG 1: 44.4, IG 2: 54.5%, CG: 65.2  ***Age***: mean (SD)  IG 1: 66.1 (6.21), IG 2: 63.5 (3.39), CG: 62.3 (3.07) | The study assessed the effects of a 5-week behavioral program following participation in a 6-week exercise program (IG 1). IG 2 received exercise program only, CG received no intervention. | Objective: Change in pedometer assessed step counts from baseline to 12 and 24 months | Age^a^ | NA |
| Baker et al. (2007) [2] | Australia | RCT, IG *n*^c^=20,  CG *n*=18 | *n*=76  ***Gender/sex***: % female  IG: 60.0, CG: 66.6  ***Age:*** mean (SD)  IG: 78.3 (7.1), CG: 74.7 (4.3) | The study tested the feasibility and efficacy of 10-week supervised exercise intervention compared to a waiting-list control group. | Self-report: Change in PASE score (habitual PA) from baseline to 10 weeks | Age^a^ | NA |
| Capodaglio et al. (2007) [3] | Italy | Quasi experimental study, IG *n*^d^=23,  CG *n*=15 | *n*=38  ***Gender/sex***: % female  IG: 52.2, CG: 46.7  ***Age:*** mean (SD)  IG: 76.6 (3.8), CG: 77.7 (3.1) | This study determined the impact of a 1-year mixed strength-training program (hospital-based exercise classes and home sessions) compared to a no-intervention control group. | Self-report: Change in AA3, intensity classes, MDEE (Paquap® questionnaire) over time | NA | Gender/sex |
| Chao et al. (2012) [4] | China | RCT, 1 IG *n*^d^=957,  CG *n*=1005 | *n*=1,962  ***Gender/sex:*** % female  IG: 52.0, CG: 53.1  ***Education:*** % illiteracy/primary school/university  IG: 10.3/19.1/59.4/11.2, CG: 8.7/17.6/62.9/10.9  ***Age:*** mean (SD)  IG: 69.81 (6.71), CG: 69.40 (7.04)  ***Marital status***: % married/divorced or widowed  IG: 84.8/15.2, CG: 83.5/16.5  ***Living situation:*** % living alone /with husband or wife/ with children  IG: 6.7/48.8/44.5, CG: 7.7/50.7/41.6 | This study evaluated the impact of a18-month community-based health management program compared to a usual-care control group. | Self-report: Change in PA (min/week) from baseline to 18 months | NA | NA |
| Clare et al. (2015) [5] | UK | Pilot RCT, IG 1 *n*^d^=21, IG2 *n*=22, CG *n*=27 | *n*=75  ***Race/ethnicity:*** % white British or Irish  GS, GM, IC:100.0  ***Occupation:***  *a)* % retired or unemployed (vs. employed)  IG1: 79.1, IG2: 66.7, CG: 78.6  b) % unskilled/partly skilled/skilled manual/skilled non-manual/managerial and technical/professional  IG1:16.7/16.7/0/16.7/41.7/8.3, IG2: 4.2/12.5/20.8/29.2/25.0/8.3, CG: 7.4/11.1/7.4/37.0/37.0/0  ***Gender/sex***: % female  IG1: 95.8, IG2: 79.2, CG: 85.2  ***Education:***  a) mean years (SD)  IG1:13.79 (3.18), IG2: 13.58 (2.68), CG: 12.70 (2.91)  b) % no formal qualifications/secondary school/vocational training/university degree/higher degree  IG1:16.7/29.2/12.5/20.8/20.8, IG2: 29.2/33.3/8.3/20.8/8.3, CG: 44.4/22.2/7.4/18.5/7.4  ***SES:*** mean (SD) (range: 0-10; perceived SES)  IG1: 6.54 (1.95), IG2: 6.54 (1.95), CG: 6.51 (1.71)  ***Income:*** % non/moderate/high material deprivation  IG1: 29.2/54.2/16.7, IG2: 8.3/62.5/29.2, CG: 37.0/44.4/18.5  ***Social capital:*** % poor (vs. good)  IG1: 62.5, IG2: 66.7, CG: 70.4  ***Age:*** mean (SD)  IG1: 67.50 (7.66), IG2: 68.21 (7.92), CG: 70.22 (7.77)  ***Marital status:*** % single/married/divorced/cohabiting  IG1: 12.5/45.8/12.5/25.0/4.2, IG2: 12.5/58.3/4.2/16.7/8.3, CG: 51.9/7.4/37.0/3.7  ***Living situation***: % living alone (vs. living with others)  IG1: 33.3, IG2: 25.0, CG: 44.4 | This study examined the feasibility and acceptability of a goal-setting intervention aimed at promoting healthy aging and reducing risk of dementia. IG1 had a goal-setting interview. IG2 had the goal-setting followed by telephone mentoring. CG group had an information interview. The intervention was delivered in the context of a community Agewell Center. | Self-report: Change in PASE score from baseline to 12 months | NA | NA |
| Croteau et al. (2007) [6] | USA | Experimental repeated measures study,  IG *n*^c^=79, CG *n*=68 | *n*=147  ***Gender***: % female  total sample: 78.8  ***Age:*** mean (SD)  total sample: 72.9 (8.8) | This study investigated the effect of a 12-week pedometer-based intervention (“A Matter of Health”) including counseling, pedometer usage, and self-monitoring. | Objective: Change in pedometer assessed daily step counts from baseline to 12-weeks. | Age | NA |
| Fernández-Ballesteros et al. (2005) [7] | Spain | Quasi-experimental pre-post with control group, IG1 (no PA data) *n*^c^=10, IG2 *n*=36, CG *n*=25 | *n*=88  ***Gender/sex***: % female  IG1: 92.3, IG2: 83.7, CG: 77.4  ***Age:*** mean (SD)  IG1:79.3 (7.6), IG2: 69.9 (6), CG: 74.2 (6.8)  ***Education***: % illiterate/not completed basic education/ elementary certificate/high school certificate/technical education/university studies  IG1: 0/53.8/15.4/23.1/7.7/0, IG2: 0/17.9/20.5/25.6/17.9/17.9, CG: 3.6/21.4/32.1/14.3/0/28.6  ***Marital status:*** % single (vs. married)  IG1: 0, IG2: 32.6, CG: 43.3 | This study examined the effectiveness of a psychosocial multimedia program (“Vivir con Vitalidad-M” (Vital Ageing-M), aimed at promoting Successful Ageing. IG1 (consisting of volunteers living in a residence for the elderly; no data on PA) and IG2 (consisting of people attending a senior center) received the program. CG received no intervention. | Self-report: Change in amount of exercises done from pre- to posttest. | NA | NA |
| Frosch et al. (2010) [8] | USA | Pilot two-group quasi-experimental study,  IG1 *n*^d^=63, IG2 *n*=53 | *n*=116  ***Race/ethnicity:*** % African American/Caucasian/Latino  IG1: 93.7/0.0/4.8, IG2: 19.6/58.8/7.8  ***Gender/sex:*** % female  IG1: 83.9, IG2: 71.7  ***Education:*** % > High school education  IG1: 50.0, IG2: 92.5  ***Income:*** % < $35,000  IG1: 63.6, IG2: 46.0  ***Age:*** mean (SD)  IG1: 70.6 (7.7), IG2: 73.6 (8.3)  ***Marital status***: % widowed  IG1: 44.1, IG2: 32.0 | This study evaluated the effect of a 12-week activation intervention delivered in community senior centers to improve health outcomes for chronic diseases. The intervention consisted of group screenings of video programs followed by moderated discussions. One senior center (IG1) was assigned to an encouragement condition (incentive) to increase participation. | Self-report: Change in number of minutes of walking and vigorous PA/week from baseline to 6 months. | Gender/sex^a,^  Age^a^ | NA |
| Geller et al. (2012) [9] | Hawai | RCT, IG1 *n*^c,d^=12,  IG2 *n*=9 | *n*=21  ***Race/ethnicity:*** Japanese/Filipino/Caucasian/Native American/Native Hawaiian/Hispanic/Others  Entire sample: 5/4/4/1/1/1/5  ***Gender/sex:*** % female  Entire sample: 76  ***Education:*** % High school graduates  Entire sample: 80  ***Age:*** mean (SD)  Entire sample: 72.24 (11.84)  ***Marital status***: % single or widowed  Entire sample: 95 | This study tested the efficiency and effectiveness of a single-day decisional balance sheet program aimed at increasing PA or fruit and vegetable intake. | Self-report: Change in average daily PA (IPAQ short form) from baseline to 2-weeks. | NA | NA |
| Gellert et al. (2013) [10] | Germany | RCT, IG1 *n*^c,d^=186,  IG2 *n*=200 | *n*=386  ***Occupation:*** % retired  Entire sample: 77.0  ***Gender/sex:*** % female  Entire sample: 50.3  ***Education:*** % higher education /finished 9^th^ grade/other  Entire sample: 67.5/23.1/1.4  ***Age:*** mean (SD)  Entire sample: 66.6 (4.8)  ***Marital status:*** % married/divorced/widowed/single  Entire sample: 66.1/17.5/ 10.8/5.6 | This study examined the effects of an age-tailored intervention (IG1) fostering present orientation and emotion focus and strategies of selection, optimization, and compensation, compared to an age-neutral intervention (IG2) aimed at enhancing planning and self-efficacy only. | Self-report: Change in frequency of physical exercise/sports and PA that had the goal of covering distances/transportation (based on German PAQ-50+). | Gender/sex, Age | NA |
| Grahn Kronhed et al. (2006) [11] | Sweden | Quasi-experimental study,  IG1 *n^d^*=141,  IG2 *n*=58,  CG *n*=86 | *n*=386  ***(Age:***  All persons aged ≥65years (no further specified))  ***Marital status:*** % living with partner  Entire sample: 63-66 | This study explored whether combined population-based (health education of personnel working at nursing homes and municipal home-help service units, associations for retired persons, study circles and sport clubs; posters; discussions in public seminars, local press, TV; balance training, walking groups, pamphlets, marketing of sturdy shoes, shoe spikes, and the importance of good lighting) and individual interventions (personal letter including a health profile and advice) directed at risk factors for osteoporosis and falls result in behavioral changes. IG1 received both interventions, IG2 received the population based intervention only. CG received no intervention. | Self-report: Difference in PA level (“low”, “moderate”, “high”) between groups at 5-years follow-up. | NA | NA |
| Greaney et al. (2008) [12] | USA | RCT, IG *n*^d^=470,  CG *n*=496 | *n*=966  ***Race/ethnicity: %*** White/Black/ Portuguese-Cape Verdean/Other  IG: 78.4/2.2/13.0, CG: 77.0/2.4/14.4/6.1  ***Gender/sex:*** % female  IG:72.8, CG: 70.4  ***Education:*** mean years (SD)  IG: 12.9 (2.7), CG: 12.9 (3.1)  ***Age:*** mean (SD)  IG:75.2 (6.7), CG: 74.7 (6.6) | As part of the “SENIOR Project”, this study examined the efficacy of a 12-month intervention tailored to the individual’s stage of change for exercise adoption. Tailored materials included manuals, newsletters, expert systems reports, and coaching calls. The 3 intervention groups ((1) increasing fruit and vegetable consumption, 2) increasing exercise, 3) increasing both) and the no-intervention control group were combined to two groups for the purpose of this study. | Self-report: Change in YPAS score from baseline to 24-months. | Gender/sex, Age | NA |
| Gudlaugsson et al. (2012) [13] | Iceland | RCT (cross-over),  IG *n*^c^=48/45,  CG *n*=58/50 | *n*=117  ***Gender/sex:*** % female  IG: 55.4, CG: 52.5  ***Age:*** mean (SD)  IG: 80.8 (4.7), CG: 78.3 (4.1) | This study evaluated the long-term effects of a 6-month multimodal training intervention consisting of daily endurance (e.g., walking) and twice-a-week strength training (fitness center) supported by seven lectures. | Objective: Difference in progression over time in accelerometer assessed counts /minute between groups. | Gender/sex, Age | NA |
| Gudlaugsson et al. (2013) [14]  See Gudlaugsson et al. (2012) |  |  |  |  |  |  |  |
| Harari et al. (2008) [15] | UK | RCT, IG *n*^c^=872,  CG1 *n*=989,  CG2 *n*=441 | *n*=2,006  ***Place of residence***: mean Townsend score) (SD) (higher scores denote higher social deprivation)  IG: 1.00 (2.9), CG1: 0.86 (2.9)  ***Gender/sex:*** % female  IG: 56.0, CG1: 52.9  ***Age:*** mean (SD)  IG1:74.7 (6.3), CG2: 74.2 (6.0) | This study reported on the effects of a Health Risk Appraisal for Older Persons questionnaire leading to computer-generated individualized written feedback to participants and GPs, integrated into practice IT systems on preventative-care. All primary care staff received training in preventative health in older people, i.e., CG1 participants received care from educated providers. | Self-report: Difference in PA (numbers and percentages of individuals reporting ≥ 5 times/week, and ≥ 3 times/week moderate to strenuous PA) between groups at 1-year follow-up. | Place of residence^b^, Gender/sex^b^, Age^b^ | NA |
| Harris et al. (2015) [16] | UK | Cluster RCT,  IG *n*^c^=137, CG *n*=136 | *n*=298  ***Place of residence***: % most /middle/least deprived  IG: 9/15/76, CG: 11/19/70  ***Race/ethnicity: %*** White  IG: 99, CG: 98  ***Occupation:*** % retired  IG: 63, CG: 55  ***Gender/sex:*** % female  IG: 54, CG: 53  ***Education:*** ≤16 years old/ 17-18 years old/19+ years old finished full-time education  IG: 46/17/37, CG: 37/14/49  ***Income:***  a) % receiving occupational pension  IG: 77, CG: 74  b) % difficulty in paying bills (ever)  IG: 12, CG: 10  ***Age:*** % 60-54/65-69/70-75  IG: 27/41/32, CG: 47/30/24  ***Marital status:*** % married/widowed/divorced or separated/single  IG: 82/8/7/3, CG: 80/6/10/5 | This study assessed the effects of a 3-month complex intervention including 4 individually tailored primary care nurse-delivered PA consultations, pedometer step-count and accelerometer PA intensity feedback, and individual PA diary plan. | Objective: Differences in change in accelerometer assessed average daily step-counts from baseline to 3 months between groups. | Gender/sex, Age | Gender/sex, Age |
| Hsu et al. (2010) [17] | China | Quasi-experimental design, IG *n*^d^=45, CG1*n*=214, CG2 *n*=255 | *n*=514  ***Race/ethnicity: %*** Mignan/Hakka/Mainlanders  IG: 24.4/68.9/6.7, CG1: 30.4/63.6/6.1, CG2: 34.1/58.4/7.5  ***Gender/sex:*** % female  IG: 51.1, CG1: 56.5, CG2: 51.0  ***Education:*** % illiterate/Elementary school or informal education/Junior high school+  IG: 17.8/48.9/33.3, CG1: 29.4/53.3/17.3, CG2: 33.7/52.9/13.3  ***Age:*** % 65-69/70-74/75-79/80+  IG: 42.2/33.3/15.6/8.9, CG1: 27.6/27.5/23.8/21.0, CG2: 28.6/20.8/28.2/22.4  ***Marital status:*** % no spouse (vs. spouse)  IG: 22.2, CG1: 42.1, CG2: 37.6 | This study evaluated the process and effects of a half-day community-based aging intervention program including education (lectures, food-recognition game, PA demonstration, and protocols) on nutrition, dietary behavior, and physical activities and phone calls during a 12-week period. CG1 comprised nonparticipants in the intervention communities, CG2 comprised residents in the control communities. | Self-report: Change in regular exercise (doing exercise for at least 30 min 3x/week) over time and differences in change between groups. | Gender/sex, Education, Age,  Marital status | NA |
| Kahlbaugh et al. (2011) [18] | USA | RCT, IG *n*^d^=16,  CG1 *n*=12, CG2 *n*=7 | *n*=36  ***Race/ethnicity:***  Entire sample: “predominantly white”  ***Gender/sex:*** % female  Entire sample: 88.9  ***Education:***  Entire sample: “predominantly had at least a high school degree”  ***Age:*** mean (SD)  Entire sample: 82 (9.8)  ***Marital status:***  Entire sample: “predominantly widowed” | This study investigated the effects of playing Wii bowling with a social partner once a week for 1 hour for a 10-week period (IG) compared to watching TV with a social partner but not playing Wii (CG1), and a neither playing Wii nor watching TV (CG2). | Self-report: Differences in changes in weekly activity score over time between groups. | NA | NA |
| Kim et al. (2011) [19] | USA | RCT with repeated measures, IG *n*^d^=48,  CG *n*=45 | *n*=93  ***Race/ethnicity: %*** African American/Asian/White/Latino/Other  IG: 43.75/29.17/16.67/8.33/2.08, CG: 33.33/51.11/13.33/2.22  ***Gender/sex:*** % female  IG: 54.17, CG:62.22  ***Age:*** mean (SD)  IG: 70.56 (8.73), CG: 70.93 (8.94) | This study examined the effects of a 6-week  guided relaxation and exercise imagery intervention compared to a placebo control group (audio CD with two relaxation tracks and advise to listen to music of own choice). | Self-report: Differences in change in total MET scores (leisure-time exercise for at least 20 min; LTEQ) from baseline to 6 weeks between groups. | NA | NA |
| Kimura et al. (2013) [20] | Japan | Cluster RCT (crossover), IG *n*^c^=57, CG *n*=35 | *n*=92  ***Gender/sex:*** % female  IG: 84.2, CGl: 77.1  ***Age:*** mean (SD)  IG: 84.2 (5.9), CGl: 74.3 (5.0) | This study examined the effectiveness of a 3-month social program (Sumida TAKE10!) held at community centers aimed at introducing and promoting PA in the home at each individual’s pace and helping to maintain good dietary habits by keeping self-check sheets. | Self-report: Change in frequency of walking, stretching and muscle strengthening from baseline to 3-months and differences in change between groups. | NA | NA |
| Knight et al. (2014) [21] | Canada | RCT, IG1 *n*^d^=15,  IG2 *n*=14, IG3 *n*=16 | *n*=45  ***Gender/sex:*** % female  IG1: 46, IG2: 64, IG3: 56  ***Age:*** mean (SD)  IG1: 63 (5), IG2: 63 (4), IG3: 62 (4) | This study tested the effects of a 12-week mHealth intervention with tailored PA prescription targeting changes to either daily exercise (IG1), sedentary behavior (IG2), or both (IG3). | Objective: Change in pedometer assessed steps/day over time. | NA | NA |
| Kullgren et al. (2014) [22] | USA | RCT, IG1 *n*^c^=20,  IG2 *n*=22, IG3 *n*=25, CG *n*=25 | *n*=92  ***Race/ethnicity: %*** White non-Hispanic/Black non-Hispanic/Other non-Hispanic/Hispanic  IG1: 95, IG2: 95.5/0/4.5/0, Combined: 91.7/8.3/0/0, Control: 92/0/0/8  ***Occupation:*** % Employed for wages/self-employed/Homemaker/retired  IG1: 10/10/10/70, IG2: 22.7/4.5/0/ 72.7, IG3: 12.5/16.7/4.2/66.7, CG: 0/12/4/83  ***Gender/sex:*** % female  IG1: 75, IG2: 72.7, IG3: 62.5, CG: 72  ***Education:*** less than college graduate/Postcollege degree  IG1: 15/15/70, IG2: 9.1/18.2/72.7, IG3: 4/40/65, CG: 40/20/40  ***Income:*** % <$50,000/50,000<$100,000/≥100,000$  IG1: 30/45/25, IG2: 28.6/52.4/19, IG3: 20.8/54.2/25, CG: 40.9/40.9/18.2  ***Age:*** mean (SD)  IG1: 72.4, IG2: 71.9 (5.6), IG3: 71.9 (5.8), CG: 71.5 (5.1)  ***Living situation:*** mean (SD) household residents  IG1: 2.0 (1.3), IG2: 1.9 (0.6), IG3: 1.9 (0.4), CG: 1.7 (0.5) | This study tested the effects of financial incentives (lottery entry when meeting walking goals) (IG1), peer networks (online message board) (IG2), and their combination (IG3) delivered through eHealth technologies compared to a control group receiving pedometers, goal-setting, and regular feedback on goal attainment only (CG). | Objective: Differences in proportion of days walking goals were met during the 16-week intervention and 8-week follow-up between groups. | NA | NA |
| Kwon (2015) [23] | South Korea | RCT, IG *n*^d^=43,  CG *n*=46 | *n*=89  ***Occupation:*** % not occupied (vs. occupied)  IG: 86.0, CG: 93.5  ***Gender/sex:*** % female  IG: 53.5, CG: 65.2  ***Religion***: % None/Buddhist/Protestant/Roman catholic  IG: 27.0/34.9/7.0/30.2, CG: 23.9/37.0/15.2/23.9  ***Education:*** % ≤Elementary school/Middle school/≥High school  IG: 25.6/23.3/51.2, CG: 23.9/28.3/47.8  ***Income:*** % 0~499,999 won/month (vs. ≥500,00 won/month)  IG: 37.2/, CG: 52.2  ***Age:*** % 65-69/70-79/ ≥80  IG: 48.8/41.9/9.3, CG: 32.6/63.1/4.3  ***Marital status:*** % married (vs. divorce/widowed)  IG: 72.1, CG: 56.5  ***Living situation:*** % living alone/with spouse/with family  IG: 23.3/55.8/20.9, CG: 32.6/45.7/21.7 | This study investigated the effects of a 4-week Wheel of Wellness counseling intervention (structured, individual counseling by nurse based on the Wheel of Wellness model). | Self-report: Change in exercise score (exercise subtask of WEL) from pre- to posttest. | NA | NA |
| Laforest et al. (2009) [24] | Canada | Quasi-experimental,  IG *n*^d^=87, CG *n*=96 | *n*=200  ***Gender/sex:*** % female  Entire sample: 84  ***Education:*** % Primary/High school/College or university  IG: 28.9/42.3/28.9, CG: 20.6/44.1/35.3  ***Income:*** % income adequate to meet basic needs  Entire sample: 68  ***Age:*** mean (SD)  IG: 73.6 (7.4), CG: 74.2 (7.4)  ***Living situation:*** % living alone  Entire sample: 58 | This study examined the 9-month impact of a 3-month community-based falls prevention program (“Stand Up!”) including balance exercises and educational components (exercise classes and home modules). | Self-report: Change in frequency of PA and energy expenditure from pre-test, to post-test and 9-months post-program and differences in change between groups. | Gender/sex, Education, Age | NA |
| Lilienthal, (2014) [25] | USA | Two group  randomly-controlled design with three Repeated measures,  IG *n*^d^=42, CG *n*=41 | *n*=86  ***Race/ethnicity:*** % White  Entire sample: 100  ***Gender/sex:*** % female  IG: 67.4, CG: 65.1  ***Education:*** % less than grade 12/ high school diploma/some college/ university degree  IG: 11.6/12.9/37.3/41.9, CG: 9.3/7.0/32.6/51.2  ***Income:*** % inadequate to meet needs  IG: 7.0, CG: 7.0  ***Age:*** mean (SD)  Entire sample: 64.48 (7.8)  ***Living situation:*** % living alone/with spouse/with an adult other than spouse  IG: 16.3/81.4/2.3, CG: 23.3/74.4/2.3 | This study examined the effects of telephone-based motivational interviewing including 4 1-hour weekly sessions tailored to fit their stage of change. Control participants received a healthy activity living guide. | Self-report: Change in weekly caloric expenditure from PA (CHAMPS-modified version: moderate-intensity PA only (MET≥3.0) from baseline, to post-treatment, and 6-months follow-up and differences in change between groups. | NA | NA |
| Mills et al. (2015) [26] | UK | RCT, IG *n^d^*=17,  CG *n*=17 | *n*=34  ***Gender/sex:*** % female  IG: 47.1, CG: 35.3  ***Age:*** mean (SD)  IG: 69 (3), CG: 68 (3) | This study examined the effects of 8-weeks of inspiratory muscle training (IG) compared to placebo (CG). | Objective & self-report: Differences in change in accelerometer-assessed counts/minute and PASE score from baseline to post-intervention between groups. | NA | NA |
| Mouton & Cloes (2015) [27] | Belgium | RCT, IG1 *n^d^*=33,  IG2 *n*=40, IG3 *n*=38, CG *n*=38 | *n*=149  ***Occupation:*** % retired  IG1: 64.6, IG2: 74.2, IG3: 70.9, CG: 69.5  ***Gender/sex:*** % female  IG1: 39.6, IG2: 32.2, IG3: 35.3, CG: 38.3  ***Education:*** % ≥ higher education level  IG1: 52.9, IG2: 43.1, IG3: 45.1, CG: 44.0  ***Income:*** % ≥ favorable  IG1: 60.3, IG2: 53.8, IG3: 52.9, CG: 54.1  ***Age:*** mean (SD)  IG1: 61.2 (6.3), IG2: 69.8 (7.4), IG3: 63.2 (5.7), CG: 66.1 (6.8) | This study examined the efficacy of a 3-month municipality-based intervention (“Move More”) available as a web-based (IG1) (PA website and monthly tailored PA advice via email), center-based (IG2) (weekly PA group sessions), and a mixed (IG3) condition. CG received no intervention. | Self-report: Change in PA level (MET min/week in leisure time, occupational, household, and transport PA at three intensities: walking, moderate, and vigorous) (IPAQ-S) from baseline to 12 months. | Occupation, Gender/sex, Education, Income, Age | NA |
| Nahm et al. (2010) [28] | USA | RCT, IG *n*^d^=115,  CG *n*=100 | *n*=245  ***Race/ethnicity:*** % White/African American/Others  IG: 89.6/8.0/2.4, CG: 92.5/6.7/0.8  ***Gender/sex:*** % female  IG: 84.8, CG: 77.7  ***Education:*** % High School or below/ Some College or College Degree/ Graduate Degree  IG: 12.0/63.2/24.8, CG: 15.0/57.5/27.5  ***Age:*** % <65/ 65-74/ 75  IG: 28.0/44.0/28.0, CG: 28.3/39.2/32.5 | This study aimed to preliminary evaluate the effectiveness of a Social Cognitive Theory-based, Structured Hip Fracture Prevention Website (learning modules and moderated discussion board) compared to a conventional Hip Fracture Prevention Website (no structured abstracted content, hyperlinks only). | Self-report: Change in exercise (exercise dimension of the YPAS) from baseline to 3 months-follow-up and differences in change between groups. | NA | Race/ethnicity, Gender/sex, Age |
| Notthoff & Carstensen (2014) [29] | USA | RCT, IG1 *n*^d^=115,  IG2 *n*=100 | *n*=59  ***Race/ethnicity:*** % European American/ African American/Asian American/other or mixed ethnicity, no ethnicity  Entire sample: 89.9/1.7/5.1/3.4  ***Gender/sex:*** % female  Entire sample: 79.66  ***Education:*** mean (SD) years  IG1: 17.18 (3.01), IG2: 17.31 (3.06)  ***Age:*** mean (SD)  IG1: 75.97 (8.99), IG2: 75.62 (7.79) | This study examined the effects of six study sessions including positively- (IG1) or negatively- (IG2) framed messages conducted at a senior center delivered over 4 weeks. | Objective: Change in number of pedometer assessed steps/day. | NA | NA |
| Parisi et al. (2015) [30] | USA | RCT, IG + CG *n*=560^d^ | *n*=702  ***Race/ethnicity:*** % Black, African-American/White, European-American/Other  IG: 92/6/2, CG: 93/4/3  ***Gender/sex:*** % female  IG: 85, CG: 85  ***Education:*** % ≤ High School or Generational Educational Development (vs. ≥ Some college)  IG: 45, CG: 43  ***Income:*** % <$15,000/ >$15,000≤$35,000/>$35,000  IG: 30/35/35, CG: 29/37/34  ***Age:*** mean (SD)  IG: 67.4 (5.9), CG: 67.4 (5.8) | This study examined the impact of “real-world” volunteer engagement (“Experience Corps®” (EC)) on the frequency of participation in various lifestyle activities over a 2-year period. In the community-based EC program, teams of older adults are trained and placed in public elementary school classrooms to perform various roles (e.g., providing literacy and math support, and assisting with behavior management activities for children in kindergarten through 3rd grade) for at least 15 hours/week. CG participants were referred to a usual volunteer activity control condition. | Self-reported: Change in participation in daily activities (LAQ) at 12 and 24 months. | Gender/sex, Education, Age | NA |
| Paxton et al. (2012) [31] | Hawai | Randomized, parallel-group experimental design, IG *n*^d^=21,  IG2 *n*=22 | *n*=43  ***Race/ethnicity:*** % White/Hawaiian, Pacific Islander/Chinese/Filipino/Japanese/Portuguese/Other  IG1: 33.3/9.5/14.3/4.8/28.6/4.8/4.8,  IG2: 19.0/14.3/9.5/14.3/28.6/4.8/9.5  ***Gender/sex:*** % female  IG1: 57.1, IG2: 52.4  ***Education:*** % High school or less (vs. some college or more)  IG1: 9.6, IG2: 28.8  ***Age:*** mean (SD)  IG1: 71.5 (9.7), IG2: 68.7 (7.3) | This study examined the effects of a feasibility study based on newsletters and telephone counseling for 8-weeks on either PA (IG1) or fruit and vegetable intake (IG2). | Self-report: Change in MET-Minutes per week in mild, moderate, strenuous activity for at least 30 minutes (adapted version of the GLTEQ) over time and difference in change between groups. | NA | NA |
| Peels et al. (2013) [32] | Netherlands | Cluster RCT, IG1 *n*^c^=275, IG2 *n*=256,  IG3 *n*=214, IG4 *n*=193, CG *n*=310 | *n*=1,248  ***Gender/sex:*** % female  IG1: 52.2, IG2: 56.3, IG3: 47.2IG4: 48.2, CG: 51.0  ***Education:*** % low education (primary, basic vocational or lower general school) (vs. high: higher general secondary education, preparatory academic education, medium vocational school, higher vocational school or university)  IG1: 41.5, IG2: 51.0, IG3: 43.5, IG4: 47.4, CG: 49.5  ***Age:*** mean (SD)  IG1: 63.2 (8.3), IG2: 63.7 (8.9), IG3: 62.6 (7.2), IG4: 61.6 (7.8), CG: 64.1 (9.0) | This study examined the long-term efficacy (i.e., 8 months post-intervention) of the 4-months “Active Plus” intervention including 3 individually tailored advices available as printed basic condition, targeting psychosocial determinants of PA (IG1), printed environmental condition, targeting psychosocial and environmental determinants (IG2), a web-based version of the basic intervention (IG3), and a web-based version of the environmental intervention (IG4), compared to a waiting list control group. | Self-report: Change in weekly days of sufficient PA (≥30 min) and weekly minutes of PA (Dutch SQUASH) and differences in changes between groups. | Gender/sex, Education, Age | Gender/sex, Education, Age |
| Peels et al. (2014a) [33] | Netherlands | Cluster RCT, IG1 *n*^c^=273, IG2 *n*=252,  IG3 *n*=214, IG4 *n*=193, CG *n*=303 | *n*=2,140  ***Occupation:*** % paid job  IG1: 40.1, IG2: 43.9, IG3: 36.8, IG4: 40.2, CG: 42.8  ***Gender/sex:*** % female  IG1: 54.1, IG2: 54.7, IG3: 47.7, IG4: 48.7, CG: 50.1  ***Education:*** % low education  IG1: 43.5, IG2: 47.3, IG3: 46.1, IG4: 47.8, CG: 50.3  ***Age:*** mean (SD)  IG1: 63.1 (8.7), IG2: 64.0 (9.4), IG3: 61.8 (7.1), IG4: 60.8  (7.5), CG: 64.2 (9.5) | This study aimed to provide insight into the long-term outcomes (i.e., 5-year, 10-year and lifetime horizons) in terms of health effects and QALYs and cost-effectiveness of the “Active Plus” intervention (see Peels et al. 2013) using a computer simulation model. | Self-report: Change in weekly MET-hours in PA (Dutch SQUASH). | Gender/sex, Age | NA |
| Peels et al. (2014b) [34] | Netherlands | Cluster RCT, IG1 *n*^c^=268, IG2 *n*=236,  IG3 *n*=185, IG4 *n*=171, CG *n*=305 | *n*=1,165  ***Gender/sex:*** % female  IG1: 55.2, IG2: 57.3, IG3: 46.5, IG4: 44.4, CG: 52.8  ***Education:*** % low education  IG1: 43.0, IG2: 48.3, IG3: 41. IG4: 48.0, CG: 49.7  ***Age:*** mean (SD)  IG1: 63.2 (8.4), IG2: 63.4 (8.6), IG3: 63.0 (7.5), IG4: 61.8 (7.5), CG: 64.3 (9.1) | This study examined the short-term effects (i.e., 2 months post-intervention) of the Active-Plus intervention (see Peels et al. 2013) and studied differences in effects among various subgroups. | see Peels et al. (2013) | Gender/sex, Education, Age | Gender/sex, Education, Age |
| Golsteijn et al. (2014) [35] | Netherlands | Cluster RCT, IG1 *n*^c^=423, IG2 *n*=429,  IG3 *n*=423, IG4 *n*=432, CG *(n*=407) | See Peels et al. (2014a) | This study aimed to provide insight in the cost-effectiveness and cost-utility of the Active Plus intervention (see Peels et al. 2013). | See Peels et al. (2014a) | NA | NA |
| Pelssers et al. 2013 [36] | Belgium | Quasi-experimental study, IG *n*^d^=359),  CG *n*=113 | *n*=580  ***Gender/sex:*** % female  IG: 69.4, CG: 62.2  ***Education:*** % elementary school/middle school/high school/university college/university  IG: 10.3/46.5/31.9/9.9/1.4,  CG: 11.6/45.2/26.7/14.4/2.1  ***Age:*** mean (SD)  IG: 69.40 (7.26), CG: 70.34 (6.38)  ***marital status***: % married/widowed/divorced/single/cohabiting  IG: 63.3/26.7/1.9/6.0/2.1, CG: 73.6/23.0/0.0/2.0/1.4 | This study evaluated the effects of a 10-week structured walking intervention ("Every Step Counts!") offered as a social activity at meeting points of a community-based senior organization. The intervention prescribed pedometer-defined walks in weekly walking schedules (fitness tailored and structured in walking load). Volunteer members of local meeting points were responsible for organizing the intervention. CG was a waiting list CG. | Self-report: Change in PA score (low-, moderate, vigorous intensity and total PA; adapted version of the GLTEQ) from baseline to 10-weeks follow-up. | Gender/sex, Education, Age,  Marital status | NA |
| Pérula et al. 2012 [37] | Spain | Cluster RCT, IG *n*^c^=133, CG *n*=271 | *n*=404  ***Gender/sex:*** % female  IG: 59.4, CG: 50.2  ***SES***: % I (highest)/II/III/IV/Iva/IVb/V (lowest)  IG: 0.0/1.5/2.3/36.1/6.8/53.4,  CG: 0.0/1.8/3.0/22.5/8.1/64.6  ***Age:*** mean (SD)  IG: 76.30 (3.85), CG: 76.46 (4.62)  ***Marital status***: % married/widowed/separated/single  IG: 86.4/25.6/2.3/3.8, CG: 73.8/23.2/0.0/3.0 | This study examined the effects of a multifactorial intervention program to prevent falls (individual advice, information leaflet, physical exercise workshop, home visits) (IG) compared with a brief intervention (brief individual advice and information leaflet) (CG). | Self-report: differences in walking (≥210 min/week) (Spanish IPAQ) at month 12 between groups. | NA | NA |
| Poulsen et al. (2007) [38] | Danmark | Prospective controlled randomised follow-up study, IG *n*^c^=997,  CG *n*=916 | *n*=1,913  ***Gender/sex:*** % female  Entire sample: 54.1  ***Age:*** % 70 years/80 years  Entire sample: 76.3/23.7 | This study investigated whether preventive home visits as part of daily routine in primary care are associated with change or stability in PA, and whether a 3-year educational program of home visitors influences stability and change in PA. IG communities received the educational intervention, control communities conducted the preventive home visits program their own way. | Self-report: Stability and change in PA from baseline to 4.5-years follow-up. | Gender/sex, Age | Gender/sex, Age |
| Solberg et al. (2014) [39] | Norway | Follow up of a RCT, IG1 *n*^d^=19, IG2 *n*=20, IG3 *n*=23 | (*n*=62):  ***Gender/sex:*** % female  IG1: 68, IG2: 55, IG3: 61  ***Education:*** % college education  IG1: 42, IG2: 37, IG3: 61  ***Age:*** mean (SD)  IG1: 73.7 (3.8), IG2: 73.7 (3.9), IG3: 75.8 (6.7)  ***Living situation:*** % living  IG1: 58, IG2: 40, IG3: 44 | This study aimed to investigated the long-term effects (i.e., 12-months post-intervention) of a 4-month exercise intervention, as well as to determine whether baseline well-being and PA values, and pre-intervention motivation moderate the intervention effects. Three intervention conditions were compared: endurance training (Nordic walking, aerobics, hiking) (IG1), functional training (circuit training) (IG2), strength training (traditional exercises) (IG3). | Self-report: Change in PA from baseline to 16 months | Gender/sex^a^, Education^a^, Age^a^,  Living situation^a^ | NA |
| Tan et al. (2006) [40] | USA | RCT, IG *n*^c^=59,  CG *n*=54 | *n*=113  ***Race/ethnicity:*** % African-American (vs. Other)  IG: 97, CG: 96  ***Gender/sex:*** % female  IG: 92, CG: 96  ***Education:*** % ≤ High School or less  IG: 83, CG: 85  ***Income:*** % <$15,000 per year  IG: 65, CG: 74  ***Age:*** % 59-65/66-70/71-75/<75  IG: 30/25/37/9, CG: 32/46/18/4  ***Marital status***: % married  IG: 24, CG: 23 | This study reports on an 8-month pilot RCT investigating the effects of the Baltimore **“**Experience Corps® Program” (see Parisi et al. 2015). | Self-report: Change in PA (modified MLTPAQ; Paffenberger physical activity questionnaire) after 4-8 months. | Race/ethnicity, Gender/sex, Education, Age | NA |
| Thomas et al. (2012) [41] | China | Cluster RCT, Total sample *n*^c^=399; IG1*n*=204, CG1 ^c^=195, IG2 *n*=193, CG2 *n*=206 | *n*=399  ***Gender/sex:*** % female  IG1: 63.2, CG1: 69.2  IG2: 65.3, CG2: 67.0  ***Education:*** % lower than primary school/ primary school/secondary school or higher  IG1: 22.1/48.0/29.9, IG1: 28.2/50.3/21.5  IG2: 20.7/54.9/24.4, CG2: 29.1/43.7/27.2  ***Age:*** mean (SD)  IG1: 71.3 (5.6), IG1: 73.0 (6.3)  IG2: 71.7 (5.7), CG2: 72.4 (6.3) | This study assessed the effects of pedometry and buddy support. Buddy peer support participants were given instructions on how to enlist support and walking partners, and were asked to reach 30 min of moderate PA, 3-5x/week with a partner. Pedometry participants were asked to increase daily number of steps by 3500 steps (3-5x/week k) using a gradual exercise plan. All intervention participants received monthly telephone calls (during 6 months), and monthly group-based face-to-face meetings. Participating community centers were randomly allocated to 1) pedometry and buddy, 2) pedometry and no buddy, 3) no pedometry and buddy, and 4) no pedometry and no buddy. IG1 comprised all individuals randomized to groups 1 or 2, CG1 all individuals randomized to groups 3 or 4, IG2 all individuals randomized to groups 1 or 3, CG2 all individuals randomized to groups 2 or 4. | Self-report: Change in level of PA energy expenditure (IPAQ-S) from baseline to 12-months follow-up. | Gender/sex, Age | NA |
| Van Stralen et al. (2009a) [42] | Netherlands | Cluster RCT, IG1 *n*^d^=444, IG2 *n*=481,  CG *n*=486 | *n*=1,1411  ***Occupation:*** % employed  Entire sample: 47  ***Gender/sex:*** % female  Entire sample: 57  ***Education:*** % low (primary, basic vocational, or lower general school)  Entire sample: 48  ***Age:*** mean (SD)  Entire sample: 64 (8.6)  ***Marital status***: % having a partner  Entire sample: 81 | This study investigated the efficacy of two tailored physical activity interventions among subgroups (i.e., complying or not complying with the guideline at baseline) 2-months post-intervention compared to a waiting-list control group (CG). A further aim was to conducted a process evaluation to test exposure and appreciation of the interventions. Both interventions consisted of 3 tailored letters delivered over 4 months. IG1 participants received an intervention targeting psychosocial determinants, IG2 participants received an intervention additionally targeting environmental determinants (i.e., information on leisure walking, cycling, and sports opportunities in the neighborhood; access to an e-buddy system and e-forum). | Self-report: Change in total weekly days of PA (Dutch SQUASH), compliance with PA guideline of 30 min of moderate PA /day on at least 5 days/week and self-rated PA level from baseline to 3-and 6-months follow-up. | Occupation,  Education, Age | NA |
| Van Stralen et al. (2009b) [43] | Netherlands | Cluster RCT, IG1 *n*^d^=444, IG2 *n*=481,  CG *n*=486 | See van Stralen (2009a) | This study aimed to explore the working mechanisms of the intervention additionally targeting environmental determinants described in van Stralen et al. 2009a. | Self-report: Change in total weekly days of PA and weekly minutes of cycling and of sport (Dutch SQUASH) from baseline to 6-months follow-up. | Occupation,  Gender/sex, Education, Age | NA |
| Van Stralen et al. (2010) [44] | Netherlands | Cluster RCT, IG1 *n*^c^=418, IG2 *n*=450,  CG *n*=458 | *n*=1,326  ***Gender/sex:*** % female  IG1: 58, IG2: 55, CG: 57  ***Education:*** % low  IG1: 52, IG2: 42, CG: 52  ***Age:*** % ≥65  IG1: 39, IG2: 41, CG: 42  ***Marital status***: % having a partner  IG1: 81, IG2: 79, CG: 82 | This study aimed to conduct in-depth analysis on the long-term efficacy (i.e., 8-months post-intervention) of two tailored PA interventions (see van Stralen et al. 2009a). A further aim was to provide further insight into possible moderators (demographic, health-related and psychosocial factors) of the effects of the two intervention conditions. | Self-report: Change in total PA, transport walking and cycling, leisure walking and cycling, and sports (Dutch SQUASH) from baseline to 12-months follow-up. | Gender/sex, Education, Age, Marital status | Gender/sex, Education, Age, Marital status |
| Van Stralen et al. (2011) [45] | Netherlands | Cluster RCT, IG1 *n*^d^=428, IG2 *n*=455,  CG *n*=465) | *n*=1,348  ***Occupation:*** % employed  Entire sample: 47  ***Gender/sex:*** % female  Entire sample: 57  ***Education:*** % low (primary, lower vocational, preparatory vocational or medium general secondary school)  Entire sample: 48  ***Age:*** mean (SD)  Entire sample: 64 (8.6)  ***Marital status***: % having a partner  Entire sample: 81 | This study examined the long-term efficacy (i.e., 8-months post-intervention) of the interventions described in van Stralen et al. 2009a and potential psychosocial and environmental mediators. | Self-report: Change in total weekly days and total weekly minutes) (Dutch SQUASH) from baseline to 12 -months follow-up. | Gender/sex, Education, Age,  Marital status | NA |
| Vrdoljak et al. (2014) [46] | Croatia | RCT (multicenter),  IG *n^d^*=371, CG *n*=367 | *n*=738  ***Occupation:*** % housewife/worker or farmer/retired/manager/ other  Entire sample: 15.7/0.7/83.1/0.1/0.4  ***Gender/sex:*** % female  Entire sample: 61.4  (***Education***: Not specified)  ***Income:*** % household monthly net income considerably below the average (<414)/somewhat below the average (415-550 €)/average (551-830 €)/above the average (>831 €)  Entire sample: 30.7/31.0/21.0/17.3  ***Age:***  a) mean (SD)  Entire sample: 72.3 (5.2)  b) % 65-74/≥75 years  Entire sample: 68.2/31.8  ***Marital status***: % unmarried/married/widowed/cohabiting/ divorced  Entire sample: 4.0/63.8/29.5/0.1/2.9 | This study compared the effectiveness of programmed and intensified intervention on lifestyle changes with the usual care of GPs. Intervention GP group received training on using (non-)pharmacological intervention systematically and in accordance to the guidelines when cardiovascular risk factors were detected, a booklet on intervention, patient education flyers, and precise protocols for monitoring participants, and participated in repeated training during 12 months. CG received usual care. | Self-report: Difference in PA (complying or not complying with the guideline of 30 min of moderate PA 5x/week or 20 min of strong PA 3x/week) between groups after 18-months follow-up. | NA | NA |
| Ward Thompson et al. (2014) [47] | UK | (1) Pre-post, cross-sectional surveys, IG *n*=56 (pre), 29 (post); CG *n*=40 (pre), 32 (post); (2) Longitudinal cohort study, IG *n*=20, CG *n*=16 | ***Race/ethnicity***: % not white British  (1) IG: 24.5, 20.6; CG: 11.1, 15.7  (2) IG: 20.0, CG: 12.6  ***Gender/sex:*** % female  (1) IG: 49.0, 37.9; CG: 37.0, 34.4  (2) IG: 63.2, CG: 68.7  ***Age:*** mean (SD)  (1) IG: 75.92 (7.3), 77.0 (8.24); CG: 74.11 (7.35), 73.77 (6.3)  (2) IG: 73.84 (7.49), CG: 70.87 (4.83)  ***Living situation***: % at home alone/at home with others/ sheltered housing alone  (1) IG: 48.0/52.0/0.0, 44.8/55.2/0.0; CG: 39.1/34.8/26.1, 38.7/41.9/19.4  (2) IG: 55.0/45.0/0.0, CG: 37.5/50.0/12.5 | This study evaluated the effect of changes to the residential street environment. The environmental interventions were part of the ‘Liveable Neighbourhoods’ program promoted by Sustrans (sustainable transport charity in the UK). In ‘DIY Streets’ pilot projects, Sustrans partnered with local communities to intervene using urban and landscape design to make streets safer and more attractive. CG consisted of matched streets without intervention. | Self-report: Difference in change in frequency of outdoor visits in and time spent outdoors in relation to utilitarian and recreational walking, gardening, outdoor sports, and other outdoor activities from baseline to 2-years follow-up (3- to 6-months post-intervention) between groups. | NA | NA |
| Zidén et al., (2013) [48] | Sweden | RCT, IG1 *n*^c^=174,  IG2 *n*=171, CG *n*=114 | *n*=459  ***Gender/sex:*** % female  IG1: 66, IG2: 64, CG: 61  ***Education:*** % higher education  IG1: 19, IG2: 23, CG: 22  ***Age:*** mean/median  IG1: 85, IG2: 85, CG: 85  ***Marital status***: % married/living together  IG1: 42, IG2: 42, CG: 54 | This study investigated the long-term effects (i.e., 2-years post-test) of 2 different health-promoting interventions (“Elderly Persons in the Risk Zone). IG1 received one preventive home visit, IG2 received 4 weekly group meetings and 1 follow-up home visit. CG received no intervention. | Self-report: Difference in change in frequency of PA (performance of outdoor walks or similar physical activities) from baseline to 1- and 2-year follow-up between groups. | NA | NA |
| **Longitudinal study designs with one group pre-post design** | | | | | | | |
| Başalaniz & Atay (2014) [49] | Turkey | Quasi-experimental study, *n*^d^=32 | *n*=32  ***Gender/sex:*** 56.2% female  ***Education:*** 62.5% primary school graduates  ***Income:*** 90.6% regular income  ***Age:*** 100%65-70 years  ***Marital status***: 81.3% married | This study examined the effects of exercise recommendations supported by written (exercise booklet) and visual (movie) materials. Data collection  took place over two months. | Self-report: Change in exercise (proportion of individuals reporting having done exercise) from baseline to 2-months follow-up. | NA | NA |
| Croteau & Richeson (2006) [50] | USA | Before-and-after study, *n*^c^=76 | *n*=76  ***Gender/sex:*** 86.84% female  ***Age:*** 100% 60-90 years | This study evaluated a 4-month pedometer-based PA program (“A Matter of Health Walking Program”), including goal setting, activity selection, self-monitoring and wearing a pedometer. A further aim was to examine the effects of age on daily ambulatory activity. | Objective: Changes in pedometer assessed step scores from baseline to post-program. | NA | Age |
| Fitts et al. 2008 [51] | USA | Before-and-after study, *n*^c^=355 | *n*=355  ***Race/ethnicity:*** % 16% non-White  ***Gender/sex:*** 76% female  ***Age:*** mean 74 (SD 8.5)  ***Marital status***: 37% married | This study examined data from participants in the 12-month community-based “EnhanceWellness” program (individualized assessment and counseling for behavior modification) to determine whether improvements in disability risk factors occurred early or late (i.e., 6 or 12 months) in the enrolment period. | Self-report: Change in proportions of physically inactive individuals (infrequent or no exercise ≙ ≤4 PACE score) from baseline to 6-months and 12-months follow-up. | NA | NA |
| Fitzpatrick et al. (2008) [52] | USA | Before-and-after study, *n*^d^=418 | *n*=418  ***Race/ethnicity:*** 44% White/ 56% Black/ 1% Other  ***Gender/sex:*** 83% female  ***Education:*** mean 11 years (SD 3)  ***Age:***  a) mean 75 (SD 8)  b) 22% ≤ 69 / 49% 70-79/ 30% ≥80 years | This study evaluated the effects of a 4-month community-based PA intervention, including 16 sessions focusing on educator-led chair exercises, promotion of walking, using a pedometer, and recording daily steps. | Self-report: Change in PA (Exercise items from SDSCA and 1998 BRFSS), and pedometer-assessed step counts from baseline to 1- to 2-months post-intervention. | NA | Race/ethnicity, Gender/sex, education, age |
| Fitzsimons et al. (2013) [53] | Scotland | Before-and-after study, *n*^c^=22 | *n*=24  ***Occupation:***29.2% employed  ***Gender/sex:*** 41.7% female  ***Age:*** mean 68 years (SD 6) | This study aimed to explore the feasibility and pilot a 2-week individualised intervention incorporating feedback from an activPAL activity monitor. | Objective: Change in time spent sitting/lying, standing and stepping, total steps and sit to stand transitions (hours/day) from baseline to 2-week follow-up. | NA | NA |
| Gellert et al. (2011) [54] | Germany | Before-and-after study, *n*^c^=302 | **(*n*=302):**  ***Gender/sex:*** 48% female  ***Age:*** mean 66.5 years (SD 4.9)  ***Marital status***: 72% having a partner | This study examined the effects of social integration (having a partner also participating in the intervention vs. having a partner not participating in the intervention vs. having no partner) and exercise-specific social support in the context of a PA intervention (intervention leaflet prompting planning and self-efficacy for PA). | Self-report: Change in PA (related to work in household and garden, transportation, and exercise/sports) (adapted version of the German-PAQ-50+) between baseline and 4-weels post-intervention. | NA | Marital status |
| Hill et al. (2005) [55] | Australia | Before-and-after study, *n*^d^=19 | *n*=23  ***Gender/sex:*** 69.6% female  ***Age:*** mean 71.0 years (SD 5.6)  ***Marital status***: 60.9% married  ***Living situation***: 26.1% living alone | This pilot study evaluated the effects of a 3-months 24 form Tai Chi Quan program as well as factors associated with participation in the program. | Self-report: Change in PA level (HAP) from baseline to 3-months follow-up. | NA | NA |
| Märki et al. (2006) [56] | Switzerland | Before-and-after study, *n*^c^=21 | *n*=29  ***Gender/sex:*** 48.3% female  ***Age:*** mean 72.2 years (SD 6.1) | This feasibility study investigated the effects of systematic counseling by GPs (including stage-matched leaflets). | Self-report: Change in PA from baseline to 2-month follow-up. | NA | NA |
| Ory et al. (2010) [57] | USA | Analysis of 2 secondary databases, *n*^c^=1233 | *n*=3,092  ***Race/ethnicity:*** 30% racial/ethnic minority group  ***Gender/sex:*** 83% female  ***Education:*** 82% high school graduates  ***Income:*** 40% ≤$15,000/y  ***Age:***  a) mean 77 years  b) 15% ≥85 years | This study describes a state-wide effort to implement and disseminate an evidence-based fall-prevention program (“A Matter of Balance/Volunteer Lay Leader model”), comprising 8 weekly classes. | Self-report: Change in number of physically active days (moderate-intensity PA for at least 30 min; BRFSS survey items) from pre- to post-test. | Race/ethnicity, Gender/sex, Age | NA |
| Porter et al. (2011) [58] | USA | Before-and-after study, *n*^c^=376 | *n*=376  ***Race/ethnicity:*** 64% White/ 36% Black  ***Gender/sex:*** 82% female  ***Education:*** mean 10 years (SD 3)  ***Age:*** mean 76 (SD 8) | This study explored the relationship of a history of depression with PA and physical function before and after a the “Seniors Taking Charge of Your Health!” intervention consisting of 12 lessons (similar to the intervention described in Fitzpatrick et al. 2008). | Self-report: Change in number of days/week participating in at least 30 min of moderate PA (BRFSS) from baseline to 1- to 2-months post-intervention. | NA | NA |
| Rahe et al. (2015) [59] | Germany | study was designed as a controlled trial, but PA was assessed in IG *n*^c^=15 only | *n*=15  ***Gender/sex:*** 66.7% female  ***Education:*** mean 16.9 years (SD 4.2)  ***Age:*** mean 67.9 (SD 4.2) | This study examined whether the cognitive effects of cognitive training supplemented with PA or exercise (IG) are superior to that of cognitive training alone. The program was delivered over 6.5 weeks and was conducted in a standardized group setting. | Self-report: Change in PA (MET-scores for walking, moderate activities, vigorous activities, total MET-score, PA categories; IPAQ) from pre- to posttest and to 1-year follow-up. | NA | NA |
| Snyder et al. (2011) [60] | USA | Prospective observational study, *n*^c^=36 | *n*=36  ***Race/ethnicity:*** 88.9% White/ 11.1% Black or African  ***Gender/sex:*** 75.0% female  ***Age:*** mean 82.0 (SD 8.6) | This study examined the effects of a 4-week walking program using pedometers, goal orientation (based on individual baseline steps), and educational materials. | Objective: Change in pedometer-assessed average number of steps/day from baseline to 4 and 6 weeks. | NA | NA |
| Ståhl et al. (2013) [61] | Sweden | Before- and-after study, *n*^d^=195 | *n*=195  ***Gender/sex:*** 61% female  ***Age:*** median 78 years | This study investigated the appreciation of environmental measures (e.g., separation of pedestrians and people using bicycles and mopeds) (“Let’s go for a walk” project) and its’ effect on perceived difficulty as pedestrians and outdoor activity. | Self-report: Change in frequency of outdoor activity in the town overall and in the residential area as a pedestrian from baseline to 5-years follow-up. | NA | Gender/sex, Age |
| Teems et al. (2011) [62] | USA | Before-and-after study, *n*^c^=587 | *n*=691  ***Race/ethnicity:*** 53.7% White/ 45.6% Black/ 0.7% Other  ***Gender/sex:*** 83.9% female  ***Education:*** mean 10.5 years (SD 3.2)  ***Age:*** mean 74.7 (SD 7.8) | This study evaluated the effects a 4-months community-based intervention to reduce risk factors related to falls and fractures, consisting of 16 sessions (8 focused on prevention of falls and fractures, all including a PA component). | Self-report: Change in time of PA/day, days participating in an exercise session, and min of PA on physically active days from baseline to 1-2-month post-intervention. | NA | NA |
| Wennberg et al. (2010) [63] | Sweden | Before-and-after study, *n*^d^=244 | *n*=244  ***Gender/sex:*** 61.3% female  ***Age:***  a) mean 77  b) 60.6% 65-79/ 39.4% 80 years | This study examined the effects of removing physical barriers according to current Swedish governmental accessibility directives, and further environmental improvements. Implementation period was 5 months. | Self-report: Change in frequency of walking outside the residence during the summer months (with or without destination) from baseline to 5-months follow-up. | NA | NA |
| **Cross-sectional study designs with control group** | | | | | | | |
| Hallgrimsdottir et al. (2015) [64] | Sweden | Cross-sectional study, IG *n*^d^=358, CG *n*=288 | *n*=646  ***Gender/sex:*** % female  IG: 61, CG: 57  ***Age:*** mean (SD)  IG: 76.5, CG: 75.5  ***Living situation:*** % living alone (vs. with ≥ 1 other person)  IG: 50, CG: 41 | This study compared a study area, in which there was an intervention in the outdoor environment (see Stahl et al., 2013) 5–8 years prior to the study, with a reference area without environmental intervention. | Self-report: Frequency of walking and of activity outside the home. | Gender/sex, Age,  Living situation | Gender/sex, Age |
| Hernandes et al. (2013) [65] | Brazil | Cross-sectional study, IG *n*^c^=134, CG *n*=104 | *n*=238  ***Gender/sex:*** % female  IG: 69, CG: 69  ***Age:*** mean (SD)  IG: 76.5, CG: 75.5 | This study compared individuals who participated in community-based exercise programs with individuals who did not participate in community-based exercise programs. | Objective: Level of pedometer-assessed PA in daily life. | NA | NA |
| John-Leader et al. (2008) [66] | Australia | cross-sectional study, IG *n*^c^=166, CG n=36 | *n*=639  ***Gender/sex:*** % female  Entire sample: 63  ***Age:*** % 60+/70+  Entire sample: 91/57 | This study evaluated the reach of the 18-month multimedia campaign 'To Be Young at Heart - Stay Active Stay Independent' (SASI) as part of a multi-strategic program to reduce falls by a community intercept survey conducted in three geographically separate locations. Participants familiar with SASI were asked whether it had increased their PA. | Self-report: comparison in number and proportion of individuals reporting an increase in PA between IG and CG. | NA | NA |

**Abbreviations:** NA= Not applicable; IG = intervention group; CG = control group; PA = Physical activity; SD = Standard deviation; AA3 = aerobic activities over 3 MET intensity.; MET = metabolic equivalent ; MDEE = Mean daily energy expenditure; PASE = Physical Activity Scale for the Elderly; IPAQ = International Physical Activity Questionnaire; German-PAQ-50+ = German Physical Activity Questionnaire; YPAS = Yale Physical Activity Survey; LTEQ = Leisure Time Exercise Questionnaire; WEL = Wellness Evaluation of Lifestyle; CHAMPS = Community Health Activities Model Program for Seniors; IPAQ-S = International Physical Activity Questionnaire-Short form; LAQ = Lifestyle Activity Questionnaire; GLTEQ = Godin Leisure-Time Exercise Questionnaire; Dutch SQUASH = Dutch Short Questionnaire to Assess Health Enhancing Physical Activity; QALYs = quality-adjusted life years; MLTPAQ = Minnesota Leisure Time Physical Activity Questionnaire; SDSCA = Summary of Diabetes Self-Care Activities; BRFSS = Behavioral Risk Factor; HAP = Human Activity Profile. Surveillance System.

^a^ PROGRESS-Plus factor was considered as confounding factor, but not included in final models.

^b^ PROGRESS-Plus factor was considered as confounding factor, but in sensitivity analyses only.

^c^ The numbers reported for sample size (*n*) correspond to the number of individuals included in analysis for measuring effects on PA.

^d^ The numbers reported for sample size (*n*) correspond to the number of individuals completing the study.

^e^ The numbers reported for sample size (*n*) correspond to the number of individuals included in analyses after imputation.

**References**

1. Azizan A, Justine M, Kuan CS. Effects of a behavioral program on exercise adherence and exercise self-efficacy in community-dwelling older persons. Curr Gerontol Geriatr Res. 2013;2013:282315.

2. Baker MK, Kennedy DJ, Bohle PL, Campbell DS, Knapman L, Grady J, et al. Efficacy and feasibility of a novel tri-modal robust exercise prescription in a retirement community: a randomized, controlled trial. J Am Geriatr Soc. 2007;55(1):1-10.

3. Capodaglio P, Capodaglio Edda M, Facioli M, Saibene F. Long-term strength training for community-dwelling people over 75: impact on muscle function, functional ability and life style. Eur J Appl Physiol. 2007;100(5):535-42.

4. Chao J, Wang Y, Xu H, Yu Q, Jiang L, Tian L, et al. The effect of community-based health management on the health of the elderly: a randomized controlled trial from China. BMC Health Serv Res. 2012;12:449.

5. Clare L, Nelis SM, Jones IR, Hindle JV, Thom JM, Nixon JA, et al. The Agewell trial: a pilot randomised controlled trial of a behaviour change intervention to promote healthy ageing and reduce risk of dementia in later life. BMC Psychiatry. 2015;15:25.

6. Croteau KA, Richeson NE, Farmer BC, Jones DB. Effect of a pedometer-based intervention on daily step counts of community-dwelling older adults. Res Q Exerc Sport. 2007;78(5):401-6.

7. Fernández-Ballesteros R, Caprara MG, García LF. Vivir con vitalidad-m®: A European multimedia programme. Psychol Spain. 2005;9:1-12.

8. Frosch DL, Rincon D, Ochoa S, Mangione CM. Activating seniors to improve chronic disease care: results from a pilot intervention study. J Am Geriatr Soc. 2010;58(8):1496-503.

9. Geller KS, Mendoza ID, Timbobolan J, Montjoy HL, Nigg CR. The decisional balance sheet to promote healthy behavior among ethnically diverse older adults. Public Health Nurs. 2012;29(3):241-6.

10. Gellert P, Ziegelmann JP, Krupka S, Knoll N, Schwarzer R. An age-tailored intervention sustains physical activity changes in older adults: a randomized controlled trial. Int J Behav Med. 2014;21(3):519-28.

11. Grahn Kronhed AC, Blomberg C, Löfman O, Timpka T, Möller M. Evaluation of an osteoporosis and fall risk intervention program for community-dwelling elderly. A quasi-experimental study of behavioral modifications. Aging Clin Exp Research. 2006;18(3):235-41.

12. Greaney ML, Riebe D, Ewing Garber C, Rossi JS, Lees FD, Burbank PA, et al. Long-term effects of a stage-based intervention for changing exercise intentions and behavior in older adults. Gerontologist. 2008;48(3):358-67.

13. Gudlaugsson J, Gudnason V, Aspelund T, Siggeirsdottir K, Olafsdottir AS, Jonsson PV, et al. Effects of a 6-month multimodal training intervention on retention of functional fitness in older adults: a randomized-controlled cross-over design. Int J Behav Nutr Phys Act. 2012;9:107.

14. Gudlaugsson J, Gudnason V, Aspelund T, Olafsdottir AS, Jonsson PV, Arngrimsson SA, et al. Effects of exercise training and nutrition counseling on body composition and cardiometabolic factors in old individuals. Eur Geriatr Med. 2013;4:431-7.

15. Harari D, Iliffe S, Kharicha K, Egger M, Gillmann G, von Renteln-Kruse W, et al. Promotion of health in older people: a randomised controlled trial of health risk appraisal in British general practice. Age Ageing. 2008;37(5):565-71.

16. Harris T, Kerry SM, Victor CR, Ekelund U, Woodcock A, Iliffe S, et al. A primary care nurse-delivered walking intervention in older adults: PACE (pedometer accelerometer consultation evaluation)-Lift cluster randomised controlled trial. PLoS Med. 2015;12(2):e1001783.

17. Hsu H-C, Wang C-H, Chen Y-C, Chang M-C, Wang J. Evaluation of a community-based aging intervention program. Educ Gerontol. 2010;36(7):547-72.

18. Kahlbaugh PE, Sperandio AJ, Carlson AL, Hauselt J. Effects of playing Wii on well-being in the elderly: Physical activity, loneliness, and mood. Activ Adapt Aging. 2011;35(4):331-44.

19. Kim BH, Newton RA, Sachs ML, Giacobbi PR, Glutting JJ. The effect of guided relaxation and exercise imagery on self-reported leisure-time exercise behaviors in older adults. J Aging Phys Act. 2011;19(2):137-46.

20. Kimura M, Moriyasu A, Kumagai S, Furuna T, Akita S, Kimura S, et al. Community-based intervention to improve dietary habits and promote physical activity among older adults: a cluster randomized trial. BMC Geriatr. 2013;13:8.

21. Knight E, Stuckey MI, Petrella RJ. Health promotion through primary care: enhancing self-management with activity prescription and mHealth. Phys Sportsmed. 2014;42(3):90-9.

22. Kullgren JT, Harkins KA, Bellamy SL, Gonzales A, Tao Y, Zhu J, et al. A mixed-methods randomized controlled trial of financial incentives and peer networks to promote walking among older adults. Health Educ Behav. 2014;41 Suppl 1:43s-50s.

23. Kwon SH. Wheel of Wellness Counseling in Community Dwelling, Korean Elders: A Randomized, Controlled Trial. J Korean Acad Nurs. 2015;45(3):459-68.

24. Laforest S, Pelletier A, Gauvin L, Robitaille Y, Fournier M, Corriveau H, et al. Impact of a community-based falls prevention program on maintenance of physical activity among older adults. J Aging Health. 2009;21(3):480-500.

25. Lilienthal KR, Pignol AE, Holm JE, Vogeltanz-Holm N. Telephone-Based Motivational Interviewing to Promote Physical Activity and Stage of Change Progression in Older Adults. J Aging Phys Act. 2014;22(4):527-35.

26. Mills DE, Johnson MA, Barnett YA, Smith WHT, Sharpe GR. The effects of inspiratory muscle training in older adults. Med Sci Sports Exerc. 2015;47(4):691-7.

27. Mouton A, Cloes M. Efficacy of a web-based, center-based or combined physical activity intervention among older adults. Health Educ Res. 2015;30(3):422-35.

28. Nahm ES, Barker B, Resnick B, Covington B, Magaziner J, Brennan PF. Effects of a social cognitive theory-based hip fracture prevention web site for older adults. Comput Inform Nurs. 2010;28(6):371-9.

29. Notthoff N, Carstensen LL. Positive messaging promotes walking in older adults. Psychol Aging. 2014;29(2):329-41.

30. Parisi JM, Kuo J, Rebok GW, Xue QL, Fried LP, Gruenewald TL, et al. Increases in Lifestyle Activities as a Result of Experience Corps (R) Participation. J Urban Health. 2015;92(1):55-66.

31. Paxton RJ, Taylor WC, Hudnall GE, Christie J. Goal Setting to Promote a Health Lifestyle. Int Proc Chem Biol Environ Eng. 2012;39:101-5.

32. Peels DA, Bolman C, Golsteijn RHJ, de Vries H, Mudde AN, van Stralen MM, et al. Long-term efficacy of a printed or a Web-based tailored physical activity intervention among older adults. Int J Behav Nutr Phys Act. 2013;10:104.

33. Peels DA, Hoogenveen RR, Feenstra TL, Golsteijn RHJ, Bolman C, Mudde AN, et al. Long-term health outcomes and cost-effectiveness of a computer-tailored physical activity intervention among people aged over fifty: modelling the results of a randomized controlled trial. BMC Public Health. 2014;14:1099.

34. Peels DA, van Stralen MM, Bolman C, Golsteijn RHJ, de Vries H, Mudde AN, et al. The differentiated effectiveness of a printed versus a Web-based tailored physical activity intervention among adults aged over 50. Health Educ Res. 2014;29(5):870-82.

35. Golsteijn RHJ, Peels DA, Evers SM, Bolman C, Mudde AN, de Vries H, et al. Cost-effectiveness and cost-utility of a Web-based or print-delivered tailored intervention to promote physical activity among adults aged over fifty: an economic evaluation of the Active Plus intervention. Int J Behav Nutr Phys Act. 2014;11:122.

36. Pelssers J, Delecluse C, Opdenacker J, Kennis E, Van Roie E, Boen F. "Every step counts!": effects of a structured walking intervention in a community-based senior organization. J Aging Phys Act. 2013;21(2):167-85.

37. Pérula LA, Varas-Fabra F, Rodríguez V, Ruiz-Moral R, Fernández JA, González J, et al. Effectiveness of a multifactorial intervention program to reduce falls incidence among community-living older adults: a randomized controlled trial. Arch Phys Med Rehabil. 2012;93(10):1677-84.

38. Poulsen T, Elkjaer E, Vass M, Hendriksen C, Avlund K. Promoting physical activity in older adults by education of home visitors. Eur J Ageing. 2007;4(3):115-24.

39. Solberg PA, Halvari H, Ommundsen Y, Hopkins WG. A 1-year follow-up of effects of exercise programs on well-being in older adults. J Aging Phys Act. 2014;22(1):52-64.

40. Tan EJ, Xue QL, Li T, Carlson MC, Fried LP. Volunteering: a physical activity intervention for older adults--The Experience Corps program in Baltimore. J Urban Health. 2006;83(5):954-69.

41. Thomas GN, Macfarlane DJ, Guo BL, Cheung BMY, McGhee SM, Chou KL, et al. Health Promotion in Older Chinese: A 12-Month Cluster Randomized Controlled Trial of Pedometry and "Peer Support". Med Sci Sports Exerc. 2012;44(6):1157-66.

42. van Stralen MM, de Vries H, Mudde AN, Bolman C, Lechner L. Efficacy of two tailored interventions promoting physical activity in older adults. Am J Prev Med. 2009;37(5):405-17.

43. van Stralen MM, de Vries H, Mudde AN, Bolman C, Lechner L. The working mechanisms of an environmentally tailored physical activity intervention for older adults: a randomized controlled trial. Int J Behav Nutr Phys Act. 2009;6:83.

44. van Stralen MM, de Vries H, Bolman C, Mudde AN, Lechner L. Exploring the efficacy and moderators of two computer-tailored physical activity interventions for older adults: a randomized controlled trial. Ann Behav Med. 2010;39(2):139-50.

45. van Stralen MM, de Vries H, Mudde AN, Bolman C, Lechner L. The long-term efficacy of two computer-tailored physical activity interventions for older adults: main effects and mediators. Health Psychol. 2011;30(4):442-52.

46. Vrdoljak D, Marković BB, Puljak L, Lalić DI, Kranjčević K, Vučak J. Lifestyle intervention in general practice for physical activity, smoking, alcohol consumption and diet in elderly: a randomized controlled trial. Arch Gerontol Geriatr. 2014;58(1):160-9.

47. Ward Thompson C, Curl A, Aspinall P, Alves S, Zuin A. Do changes to the local street environment alter behaviour and quality of life of older adults? The 'DIY Streets' intervention. Br J Sports Med. 2014;48(13):1059-65.

48. Zidén L, Häggblom-Kronlöf G, Gustafsson S, Lundin-Olsson L, Dahlin-Ivanoff S. Physical function and fear of falling 2 years after the health-promoting randomized controlled trial: elderly persons in the risk zone. Gerontologist. 2014;54(3):387-97.

49. Başalaniz F, Atay E. HOW EFFECTIVE ARE EXERCISE RECOMMENDATIONS SUPPORTED BY WRITTEN AND VISUAL MATERIALS IN ELDERLY PEOPLE? Turk J Geriatr. 2014;17(4):410-6.

50. Croteau KA, Richeson NE. A matter of health: using pedometers to increase the physical activity of older adults. Activ Adapt Aging. 2005;30(2):37-47.

51. Fitts SS, Won CW, Williams B, Snyder SJ, Yukawa M, Legner VJ et al. What is the Optimal Duration of Participation in a Community-Based Health Promotion Program for Older Adults? J Appl Gerontol. 2008;27(2):201-14.

52. Fitzpatrick SE, Reddy S, Lommel TS, Fischer JG, Speer EM, Stephens H, et al. Physical activity and physical function improved following a community-based intervention in older adults in Georgia senior centers. J Nutr Elder. 2008;27(1-2):135-54.

53. Fitzsimons CF, Kirk A, Baker G, Michie F, Kane C, Mutrie N. Using an individualised consultation and activPAL feedback to reduce sedentary time in older Scottish adults: results of a feasibility and pilot study. Prev Med. 2013;57(5):718-20.

54. Gellert P, Ziegelmann JP, Warner LM, Schwarzer R. Physical activity intervention in older adults: does a participating partner make a difference? Eur J Ageing. 2011;8(3):211-9.

55. Hill K, Choi W, Smith R, Condron J. Tai Chi in Australia: Acceptable and effective approach to improve balance and mobility in older people? Australas J Ageing. 2005;24(1):9-13.

56. Märki A, Bauer GB, Angst F, Nigg CR, Gillmann G, Gehring TM. Systematic counselling by general practitioners for promoting physical activity in elderly patients: a feasibility study. Swiss Med Wkly. 2006;136(29-30):482-8.

57. Ory MG, Smith ML, Wade A, Mounce C, Wilson A, Parrish R. Implementing and disseminating an evidence-based program to prevent falls in older adults, Texas, 2007-2009. Prev Chronic Dis. 2010;7(6):A130.

58. Porter KN, Fischer JG, Johnson MA. Improved physical function and physical activity in older adults following a community-based intervention: Relationships with a history of depression. Maturitas. 2011;70(3):290-4.

59. Rahe J, Petrelli A, Kaesberg S, Fink GR, Kessler J, Kalbe E. Effects of cognitive training with additional physical activity compared to pure cognitive training in healthy older adults. Clin Interv Aging. 2015;10:297-310.

60. Snyder A, Colvin B, Gammack JK. Pedometer use increases daily steps and functional status in older adults. J Am Med Dir Assoc. 2011;12(8):590-4.

61. Ståhl A, Horstmann V, Iwarsson S. A five-year follow-up among older people after an outdoor environment intervention. Transp Policy. 2013;27:134-41.

62. Teems J, Hausman DB, Fischer JG, Lee JS, Johnson MA. Older adults attending Georgia senior centers increase preventive behaviors for falls and fractures following a community-based intervention. J Nutr Gerontol Geriatr. 2011;30(1):72-85.

63. Wennberg H, Hydén C, Ståhl A. Barrier-free outdoor environments: Older peoples’ perceptions before and after implementation of legislative directives. Transp Policy. 2010;17(6):464-74.

64. Hallgrimsdottir B, Svensson H, Ståhl A. Long term effects of an intervention in the outdoor environment-a comparison of older people's perception in two residential areas, in one of which accessibility improvements were introduced. J Transp Geogr. 2015;42:90-7.

65. Hernandes NA, Probst VS, Da Silva RA, Jr., Januário RSB, Pitta F, Teixeira DC. Physical activity in daily life in physically independent elderly participating in community-based exercise program. Braz J Phys Ther. 2013;17(1):57-63.

66. John-Leader F, Van Beurden E, Barnett L, Hughes K, Newman B, Sternberg J, et al. Multimedia campaign on a shoestring: promoting 'Stay Active - Stay Independent' among seniors. Health Promot J Austr. 2008;19(1):22-8.
